# Essential Role for *Schizosaccharomyces pombe pik1* in Septation

**DOI:** 10.1371/journal.pone.0006179

**Published:** 2009-07-09

**Authors:** Jae-Sook Park, Sarah K. Steinbach, Michel Desautels, Sean M. Hemmingsen

**Affiliations:** 1 Department of Microbiology and Immunology, College of Medicine, University of Saskatchewan, Saskatoon, Saskatchewan, Canada; 2 Department of Physiology, College of Medicine, University of Saskatchewan, Saskatoon, Saskatchewan, Canada; 3 Plant Biotechnology Institute, National Research Council Canada, Saskatoon, Saskatchewan, Canada; Dartmouth College, United States of America

## Abstract

**Background:**

*Schizosaccharomyces pombe pik1* encodes a phosphatidylinositol 4-kinase, reported to bind Cdc4, but not Cdc4^G107S^.

**Principal Findings:**

Gene deletion revealed that *pik1* is essential. In cells with *pik1* deleted, ectopic expression of a loss-of-function allele, created by fusion to a temperature-sensitive dihydrofolate reductase, allowed normal cell proliferation at 25°C. At 36°C, cells arrested with abnormally thick, misplaced or supernumerary septa, indicating a defect late in septation. In addition to being Golgi associated, ectopically expressed GFP-tagged Pik1 was observed at the medial cell plane late in cytokinesis. New alleles, created by site-directed mutagenesis, were expressed ectopically. Lipid kinase and Cdc4-binding activity assays were performed. Pik1^D709A^ was kinase-dead, but bound Cdc4. Pik1^R838A^ did not bind Cdc4, but was an active kinase. Genomic integration of these substitutions in *S. pombe* and complementation studies in *Saccharomyces cerevisiae pik1-101* cells revealed that D709 is essential in both cases while R838 is dispensable. In *S. pombe*, ectopic expression of *pik1* was dominantly lethal; while, *pik1^D709A,R838A^* was innocuous, *pik1^R838A^* was almost innocuous, and *pik1^D709A^* produced partial lethality and septation defects. The *pik1* ectopic expression lethal phenotype was suppressed in *cdc4^G107S^*. Thus, D709 is essential for kinase activity and septation.

**Conclusions:**

Pik1 kinase activity is required for septation. The Pik1 R838 residue is required for important protein-protein interactions, possibly with Cdc4.

## Introduction

Phosphoinositides are involved in signal transduction, regulation of actin cytoskeletal organization, cytokinesis, and membrane traffic in eukaryotic cells, including in yeasts [1], [2], [3], [4]. Phosphatidylinositol 4-kinases phosphorylate phosphatidylinositol (PtdIns) to produce PtdIns 4-phosphate, the precursor to other important phosphoinositides [5]. PtdIns 4-kinases have been implicated in regulation of cell division in *Saccharomyces cerevisiae* [6], [7] and *Drosophila melanogaster* [8]. Cytokinesis in *S. pombe* is a multistep process involving division site selection, contractile ring formation and contraction, membrane expansion/ingression and formation/dissolution of primary and secondary septa [9], [10], [11]. The specific aspects of cytokinesis that require PtdIns 4-kinase activity remain to be determined.

*S. cerevisiae* and *S. pombe* each appear to have 3 distinct PtdIns 4-kinases. In the budding yeast these are; Lsb6p, a type II enzyme; Stt4p, a type IIIα enzyme; and Pik1, a type IIIβ enzyme [3]. The functions of Stt4p and Pik1 are non-redundant as each is essential for viability [12], [13]. Stt4p is required for normal actin cytoskeletal organization [13]. Pik1p is required for maintenance of Golgi structures, protein secretion and endocytosis and for cytokinesis [7], [13], [14]. A conditionally lethal allele, *pik1-101*, arrests without completing cytokinesis [6], [7]. *LSB6* is non-essential [15]; however, it is partially redundant with *STT4* and is required for actin nucleation and endosome motility [15], [16].

In *S. pombe*, locus SPAC343.19 appears to encode a type II enzyme, locus SPBC577.06c appears to encode a type IIIα enzyme, and *pik1* (SPAC22E12.16c) is a putative type IIIβ enzyme. Sequence comparisons suggest that *S. cerevisiae PIK1* and *S. pombe pik1* are orthologs; although, sequence similarities outside the lipid kinase domains are weak (27% identity, compared to 53% between kinase domains). The *S. cerevisiae* enzyme is in the nucleus and associated with the Golgi [14]. Both pools of the protein are essential for cell viability [14]. The distribution of Pik1 between the nucleus and the cytosol requires association with two 14-3-3 proteins [17]. The *S. pombe* protein appears associated with the Golgi [18]. However, a nuclear localization of Pik1 in *S. pombe* has not been reported. The localization of *S. pombe* Pik1 during the cell cycle and the importance of this enzyme for cell division have yet to be determined.

*S. cerevisiae* Pik1 interacts with a number of proteins, including Frq1 [19] and Bmh1 and Bmh2 [20]. Interactions between *S. pombe* Pik1 and the corresponding homologs of the *S. cerevisiae* proteins, Ncs1, Rad25 and Rad24, have not been reported.

*S. pombe* Pik1 has been reported to interact with Cdc4 [21]. This interaction was abolished by only one of six conditionally lethal mutations of *cdc4* (*cdc4^G107S^*) [21]. Cdc4 is a contractile ring component that is required for cytokinesis [22]. The contractile ring is a transient, dynamic structure made of actin, myosins and many other associated proteins [9], [23]. The timing and site of assembly of the ring and the regulation of its contractile activity are essential for cell division [24]. One established function of Cdc4 is that of a myosin essential light chain, bound to the first IQ domain in the neck region of type II myosins [25], [26], [27]. A putative pseudo-IQ motif has been identified in Pik1, a possible interaction site with Cdc4 [21], [28].

Cdc4 also interacts with Rng2, another contractile ring component, which is required for its assembly [29]. The *rng2* gene encodes a protein related to the human IQGAP1 protein, which binds actin and calmodulin and is a potential effector for the Rho family of GTPases. Cdc4 presumably interacts with Rng2 at one or several of its 6 IQ-motifs [29]. Thus, it is possible that Cdc4 has both a structural function, as a myosin essential light chain, and regulatory functions. There is much evidence that Cdc4 has functions in addition to that of a myosin essential light chain [21], [27], [30], [31]. Six *cdc4* mutations have been identified that cause temperature-dependent failure of cytokinesis [32], [33]. Five of those alleles, with highly penetrant, conditionally lethal phenotypes, were examined for interallelic complementation in diploid cells [21]. Strikingly, three of the ten possible heterozygous diploid strains were viable at the restrictive temperature. These observations are not consistent with Cdc4 functioning only as a myosin essential light chain. Structurally, two of the mutant proteins examined (*cdc4^G19E^* or *cdc4^G107S^*) were very stable, even at the restrictive temperature, suggesting that failure of cytokinesis is due to subtle changes in structure that may impair protein interactions [34]. Furthermore, only one of the six mutations (*cdc4^R33K^*) was shown to affect actin-myosin function in sliding filament motility assays at the non-permissive temperature [30]. In addition, strains carrying either of two temperature-sensitive *cdc4* alleles (*cdc4^G19E^*, *cdc4^G107S^*) and a modified myosin that lacked both IQ domains and thereby unable to bind Cdc4, still showed lethality at the non-permissive temperature [27]. These results suggest that failure of cytokinesis at the restrictive temperature is not due to failure of interaction of these two mutant forms of Cdc4 with myosins. Finally, the cell concentration of Cdc4 has been reported to be 10-fold higher than the concentrations of type II myosins (Myo2 and Myp2) or of the myosin regulatory light chain Rlc1 [31]. This is unusual, as regulatory and essential light chains are found in equimolar amounts in the neck region of type II myosins. These observations clearly suggest that Cdc4 has functions beyond its association with myosins.

It is not known if the essential functions of *S. pombe pik1* and *S. cerevisiae PIK1* are conserved. We hypothesized that in *S. pombe*, Pik1 lipid kinase activity and possibly its Cdc4-binding activity are involved in a specific aspect of cytokinesis. To test this hypothesis, we created and characterized a number of new *pik1* alleles; including, a chromosomal *pik1* deletion allele, a conditional loss-of-function allele, a fluorescently tagged allele, and 3 point mutation alleles. The latter 3 alleles were designed to affect Pik1 lipid kinase and Cdc4-binding activities. Alleles were assessed after genomic integration or ectopic expression, and for complementation of the conditionally lethal *S. cerevisiae pik1-101* allele.

## Materials and Methods

### Strains, media, genetic and molecular biology methods

*S. pombe* strains (Table 1) were cultured in YES, ME or EMM with supplements, and transformed using lithium acetate or electroporation as described [35]. *S. cerevisiae* strains (Table 2) were cultured in synthetic minimal medium lacking leucine (SD-Leu; 0.67% nitrogen base, 2% glucose, and amino acid supplements as appropriate) in the presence or absence of 15 µM thiamine. Standard techniques for DNA manipulation were used. All recombinant sequences were verified with a model 370A automated sequencer (PE Applied Biosystems Inc.).

**Table 1:** Schizosaccharomyces pombe strains.

Strain	Genotype	Source
N2	*h^+^ leu1-32 ura4-D18 ade6-210*	P. Nurse
N3	*h^−^ leu1-32 ura4-D18 ade6-216*	P. Nurse
N250	*h^−^his3-D1 leu1-32 ura4-D18 ade6-210*	ATCC
N253	*h^+^ his3-D1 leu1-32 ura4-D18 ade6-216*	ATCC
N1185	*h^+^ cdc4^G107S^ leu1-32 ade6-210 ura4-D18*	P. Nurse
N1477	N2, pREP1	This study
N1174	N2, pREP1-*pik1*	This study
N1375	N2, pREP1-*pik1^D709A^*	This study
N1273	N2, pREP1-*pik1^R838A^*	This study
N1296	N2, pREP1-*pik1^D709A, R838A^*	This study
N1276	N1185, pREP1	This study
N1281	N1185, pREP1-*pik1*	This study
N1278	N1185, pREP1-*pik1^D709A^*	This study
N1279	N1185, pREP1-*pik1^R838A^*	This study
N1280	N1185, pREP1-*pik1^D709A, R838A^*	This study
N1550	*h^+^/h^−^ pik1/pik1::term^nmt1^::ura4 ade6-M210/ade6- M216 his3-D1/his3-D1 leu1-32/leu1-32 ura4-D18/ura4-D18*	This study
N1565	*h^+^/h^−^ pik1/pik1^D709A^::term^nmt1^::ura4 ade6-M210/ade6-M216 his3-D1/his3-D1 leu1-32/leu1-32 ura4-D18/ura4-D18*	This study
N1582	*h^+^/h^−^ pik1/pik1^R838A^::term^nmt1^::ura4 ade6-M210/ade6-M216 his3-D1/his3-D1 leu1-32/leu1-32 ura4-D18/ura4-D18*	This study
N1596	*h^+^/h^−^ pik1/pik1^D709A, R838A^ ::term^nmt1^::ura4 ade6-M210/ade6-M216 his3-D1/his3-D1 leu1-32/leu1-32 ura4-D18/ura4-D18*	This study
N1085	*gma12*-GFP::*ura4*	M. Balasubramanian MBY104
N1231	*h^+^/h^−^ pik1/Δpik1::ura4 ade6-M210/ade6-M216 leu1-32/leu1-32 ura4-D18/ura4-D18*	This study
N1113	*h^+^ Δpik1::Kan^R^ ade6-M210 leu1-32 ura4-D18*, pREP81-*pik1*	This study
N1366	*h^−^ Δpik1::ura4 ade6-M216 leu1-32 ura4-D18*, pREP41X-Ub-R-DHFR^ts^-*pik1* (referred as *pik1-td* cells)	This study
N1369	*h^+^ Δpik1::ura4 ade6-M210 leu1-32 ura4-D18*, pREP41*-2XeGFP-pik1*	This study
N1401	*h^+^ cdc25-22 leu1-32*, pREP41-2XeGFP-*pik1*	This study

**Table 2:** Saccharomyces cerevisiae strains.

Strain	Genotype	Source
N1285	*MAT a*, GAL^+^, *leu2-3,112, ura3-1, trp1-1, his3-11,15, can1-100, ade2-1*	Xiao, W.
N1255	*MAT a*, GAL^+^, *leu2-3,112, ura3-52, pik1-101*	Novick, P.
N1301	N1285, YEplac181-P*_nmt1_*-*S. pombe pik1*-T*_nmt1_*	This study
N1302	N1255, YEplac181-P*_nmt1_*-*S. pombe pik1*-T*_nmt1_*	This study
N1310	N1285, YEplac181	This study
N1311	N1255, YEplac181	This study
N1312	N1285, YEplac181-P*_nmt41_*-eGFP- *S. pombe pik1*-T*_nmt1_*	This study
N1313	N1255, YEplac181-P*_nmt41_*-eGFP- *S. pombe pik1*-T*_nmt1_*	This study
N1314	N1285, YEplac181-P*_nmt81_*-*S. pombe pik1*-T*_nmt1_*	This study
N1315	N1255, YEplac181-P*_nmt81_*-*S. pombe pik1*-T*_nmt1_*	This study
N1322	N1285, YEplac181-P*_nmt41_*- *S. pombe pik1*-T*_nmt1_*	This study
N1323	N1255, YEplac181-P*_nmt41_*-*S. pombe pik1*-T*_nmt1_*	This study
N1362	N1255, YEplac181-P*_nmt41_*-eGFP- *S. pombe pik1^D709A^*-T*_nmt1_*	This study
N1363	N1285, YEplac181-P*_nmt41_*-eGFP-*S. pombe pik1^D709A^*-T*_nmt1_*	This study
N1467	N1285, YEplac181-P*_nmt41_*-eGFP-*S. pombe pik1^R838A^*-T*_nmt1_*	This study
N1468	N1255, YEplac181-P*_nmt41_*-eGFP-*S. pombe pik1^R838A^*-T*_nmt1_*	This study

### Gene deletion

The *pik1* coding region was replaced with a *ura4* expression cassette by homologous recombination in diploid cells [36]. The diploid strain (N2 X N3) was transformed with a DNA fragment containing *ura4* flanked by 600 bp regions 5′ and 3′ to the *pik1* coding region. Diploid transformants were selected on EMM –adenine –uracil. Sequencing of strain N1231 confirmed replacement of one copy of the *pik1* coding sequence by *ura4*. N1231 spores from azygotic asci were incubated on YES at 19°C for 13 days, or at 25°C, 30°C, or 36°C for 5 days. Cells were transferred to EMM –uracil to assess for the presence of *ura4*. The presence of the *pik1* coding sequence was determined by PCR. Similarly, the *pik1* coding region was deleted in haploid cells [36] that carried pREP81-*pik1*, except a *Kan^R^* cassette was used to produce strain N1113.

### *pik1* cDNA cloning and site directed mutagenesis

A *pik1* cDNA clone was isolated by reverse transcription PCR using primers H1280 and H1285 that incorporated *Nde*I and *Bam*HI sites for cloning in pREP vectors [37], [38]. An internal *Nde*I site was removed by site-directed mutagenesis [39] using primer H1284, which introduced a silent C to T mutation at nucleotide 300 of the coding region (Genbank accession number FJ918571). The *pik1^D709A^* and *pik1^R838A^* alleles were similarly generated using primers H1340 and H1341 which introduced the mutations and restriction sites (*Pst*I and *Mfe*I, respectively) for screening (Genbank accession numbers FJ918572 and FJ918573, respectively). The double mutant *pik1^D709A, R838A^* was generated by replacing the *Bam*HI-*Age*I fragment of *pik1^D709A^* with the corresponding fragment from *pik1 ^R838A^* (Genbank accession number FJ918574). Primer sequences are available upon request.

### *pik1* allele replacement

Homologous recombination in diploid cells [36] was used to introduce missense mutations into *pik1*, producing substitution R838A, or D709A, or both. Allele replacement constructs consisted of the last 380 codons of *pik1* (*pik1* ^472–851^), the native stop codon, the *nmt1* terminator region, *ura4* gene cassette and 700 bp of genomic DNA downstream of the *pik1* coding region. As a result, the *pik1* locus was modified by the presence of *nmt1* transcription termination sequences and by the presence of a downstream *ura4* cassette. To control for these changes, the wild-type sequence was integrated into the *pik1* gene by the same method. Diploid cells (N250 X N253) were transformed, plated at 30°C on EMM –adenine –uracil. Colonies were tested for integration by colony PCR and growth on EMM –uracil. Tetrad analysis was used to establish the essentiality of each haploid *pik1* allele.

### *pik1-td* allele

The N-degron approach fuses a protein of interest to one that is conditionally unstable. The latter consists of monoubiquitin, an arginine residue, and a thermolabile dihydrofolate reductase (Ub-R-DHFR^ts^). Long-lived at 25°C, at 36°C it is rapidly degraded by the N-end rule pathway [40], [41]. The Ub-R-DHFR^ts^ coding region from pPW66R [40] was amplified by PCR as described [41] and fused, in frame, to the *pik1* cDNA coding region in pREP41X [37]. Hemizygous N1231 cells were transformed. A haploid strain carrying both pREP41X-Ub-R-DHFR^ts^-*pik1* and the disrupted chromosomal *pik1* allele (N1366, also referred to as *pik1-td*), was selected by random spore analysis. In N1366, the sole source of Pik1 is episomal.

### Fluorescently tagged *pik1* allele

The *pik1* coding region was cloned in pREP41-eGFP [42]. A second eGFP coding region was inserted in frame as an *Nde*I fragment at the *pik1* initiation codon to produce pREP41-2XeGFP-*pik1* which was used to transform hemizygous strain N1231. Haploid strain N1369 carrying both the vector and the disrupted chromosomal *pik1* allele was selected by random spore analysis.

### Synchronized cultures

To examine cell cycle dependent changes in 2XeGFP-Pik1 localization, a *cdc25-22* strain was transformed with pREP41-2XeGFP-*pik1*. Division synchrony of the resulting strain, N1401, was induced by block and release. Cells grown at 25°C in EMM –leucine –thiamine for 16 hours, were shifted to 36°C for 4 hours. Cells collected by centrifugation (5 min., 3000×*g*) were resuspended in 50 ml medium at 25°C and incubated for 220 min. Cells were sampled every 20 min. and examined immediately by microscopy to evaluate the intracellular localization of 2XeGFP-Pik1 or fixed with formaldehyde, incubated for 30 min. on a rotating wheel, washed 3 times in phosphate-buffered saline and kept at 4°C for microscopic examination [35].

### Microscopy

Cell morphology and mitotic index were examined by bright-field and fluorescence microscopy after formaldehyde fixation and Calcofluor White (Fluorescent Brightener 28, Sigma F3543-1G) and 4′6,-diamidino-2-phenylindole (DAPI) staining [35]. Cell numbers were estimated with a hemocytometer. F-actin-containing structures were visualized with FITC-phalloidin [43]. Myo2 and Pik1 immunofluorescence microscopy of methanol-fixed cells [35] used both primary antibodies and Texas red conjugated secondary antibodies at 1∶100 dilution. Rabbit Myo2 antibodies were obtained from M. K. Balasubramanian (Temasek Life Sciences Laboratory, Singapore). Rabbit Pik1 antiserum was already available [21].

An Olympus 1X70 inverted microscope with 60× 1.4NA Plan-apo objective, appropriate filter sets and a RT-Slider (SPOT) CCD camera (Carsen Scientific Imaging Group, Markham, Canada) was used for bright-field and fluorescence microscopy. Images were cropped and processed for brightness and contrast with Spot32 Advanced software.

Potassium permanganate-fixed *pik1-td* cells were examined by transmission electron microscopy as described [44], [45]. Cell preparations were done by P.A. Netto, Temasek Life Sciences Laboratory, Singapore.

### Ectopic expression of *pik1* and mutant alleles in *S. pombe*

Recombinant pREP vectors [37], [38] expressing *pik1* alleles were introduced into *cdc4* or *cdc4^G107S^* strains. Starter cultures in EMM –leucine +thiamine were incubated overnight at 30°C or 25°C (for temperature-sensitive strains). Cells were collected by centrifugation (700×*g*, 10 min.), washed 3 times in sterile distilled water, placed in 50–100 ml cultures at 10^5^ cells/ml, and incubated for 24 h. at 30°C or 25°C, + or − thiamine. Cell numbers were estimated with a hemocytometer. Cells were collected by centrifugation, and either washed and fixed for microscopic examination, or lysed with a ‘mini’ French pressure cell at 900 p.s.i. for protein estimation, immunoblotting, lipid kinase assays and ELISA.

### Colony formation assay

Starter cultures in EMM –leucine +thiamine were incubated to saturation (24–36 hours) at 30°C or 25°C. Cells were collected by centrifugation and washed 3 times in sterile distilled water. Aliquots (5 µl) from each of 4, 10-fold serial dilutions were spotted onto EMM –leucine, + or − thiamine, + phloxin B plates and incubated at 30°C for 5–6 days.

### Phospholipid kinase assay

Cells carrying *pik1* expression vectors were cultured for 24 h., + or − thiamine, at 30°C or 25°C. Cells were harvested by centrifugation (700×*g* for 5 min.), resuspended in 25 mM HEPES, pH 7.4, 10 mM MgCl_2_, and passed through a ‘mini’ French pressure cell 3 times at 900 p.s.i. Cell lysate protein content was estimated [46] and lysates were diluted to 0.05 µg–0.8 µg total protein per 50 µl assay. Wild-type and mutant strains were compared at the same time, under the same condition and at the same protein concentration. Each diluted cell lysate was incubated with 10 µCi [γ- ^32^P] ATP for 15 min. at room temperature and the reaction stopped by addition of 6 M HCl to a final concentration of 1.7 M. Lipids were extracted with three volumes of chloroform:methanol (1∶1, vol∶vol), vortexed for 10 s. and centrifuged in an Eppendorf microcentrifuge at maximum speed for 5 min. [47]. The bottom organic layer was removed and dispensed into a fresh tube. The acidic lipid fraction was further extracted with ½ volume methanol: 1 N HCl (1∶1, vol∶vol), vortexed and centrifuged as previously described. The organic phase was again retrieved and dried under N_2_ gas. The lipids were resuspended in 4 µl of chloroform:methanol (1∶1, vol∶vol) and spotted onto a Silica gel 60 thin layer chromatography (TLC) plate previously baked for 30 min. at 100°C. The TLC plate was placed in a chromatography chamber pre-equilibrated for two hours with freshly made developing solution (1-propanol∶2 M acetic acid (13.7∶7 vol∶vol)). Lipid separation was for 6–8 h. The TLC plate was dried overnight and either exposed to Kodak BioMax XAR film for 2–3 days or scanned under 10% methane in argon using a BIOSCAN AR-2000 imaging scanner for radio-TLC. WinSCAN 2D software version 1.05 was used to visualize the distribution of radioactivity on the plate. The silica carrying the radiolabelled lipid was added to Aquasol (Perkin-Elmer) for liquid scintillation counting. Data were corrected for counting efficiency and decay and expressed as disintegrations per minute (DPM). Lipid standards purchased from Avanti Polar Lipids (50 µg each of PtdIns, PtdIns(4)P, PtdIns(4,5)P_2_ and PtdIns(3,4,5)P_3_) were run in parallel and visualized using iodine vapour.

### Yeast two-hybrid assay

Assays were performed as described [48] using the leucine selectable pBI880 vector carrying the *GAL4*-DB (DNA binding domain) fused in-frame to a *cdc4* cDNA sequence, and the tryptophan selectable pBI771 vector carrying sequences encoding the *GAL4*-TA (trans-activating domain) fused in-frame to residues 507 through 851 of wild-type or mutant *pik1* alleles. Both vectors were introduced simultaneously into *S. cerevisiae* YPB2 cells using a lithium acetate procedure. Cells were selected for the presence of both plasmids by colony formation on SD –leucine – tryptophan plates at 30°C for 5 days. A positive protein interaction was then identified by colony formation at 30°C for 7–9 days on SD –leucine –tryptophan –histidine +3-amino-1′,2′,4′-triazole (3-AT). X-gal colony filter assays were also performed to confirm positive yeast two-hybrid interactions [48].

### ELISA

*S. pombe* cultures, initially at 1×10^5^ cells/ml, carrying pREP1 plasmids expressing wild-type or mutant *pik1* alleles, were incubated + or − thiamine for 24 h. at 30°C. Cell extracts were prepared using a French press ‘mini’ cell (3 passages at 900 p.s.i.) in phosphate-buffered saline (PBS) with protease inhibitors (Sigma, P8215) at 4°C. Multiwell plates were coated by incubating with purified Cdc4 protein [34] at 10 µg/ml overnight at 30°C. The wells were blocked by incubating with 2% (w/vol) powdered skim milk in PBS at room temperature for 1 h. Serial two-fold dilutions of cell lysates containing 56 µg to14 µg protein were added to the Cdc4-coated wells and incubated for 16 h. at 4°C. Wells were washed 4 times with PBS-0.01% (vol/vol) Tween 20 and incubated with a 1∶1000 dilution of rabbit anti-Pik1 serum [21] for 4 h. at room temperature followed by 1 h. at room temperature with goat anti-rabbit IgG-HRP (horseradish peroxidase) at a dilution of 1∶5000. Signal detection was with TMB (3,3′,5,5′-tetramethylbenzidine). Optical density was measured with a Molecular Devices microplate reader at wavelengths 650 nm and 450 nm, and the data acquired using Softmax Software, version 2.34.

### Complementation analysis

*S. pombe pik1* wild-type and mutant cDNA coding regions were cloned (*Nde*I – *Bam*HI) in *S. pombe* expression vectors pREP1, pREP41, and pREP81 [37], and pREP41-eGFP N [42]. The *pik1* coding regions flanked by the *nmt1* promoter and terminator sequences were then cloned (*Pst*I – *Sst*I) in *S. cerevisiae* expression vector, YEplac181 [49]. *S. cerevisiae* cells carrying *PIK1* or *pik1-101* were transformed with each of the resulting vectors using lithium acetate [50]. For colony formation assays, cells from precultures were counted and cultures were started at 1×10^5^ cells/ml at 25°C. After 18–20 h., cells were recovered by centrifugation, washed 3-times in sterile water, and resuspended at 2×10^7^ cells/ml. Aliquots (5 µl) of serial 10-fold dilutions were spotted onto SD –Leu plates and incubated at a permissive (25°C) or restrictive (37°C) temperature for 5 days.

## Results

### *pik1* is essential

**Figure 1:**
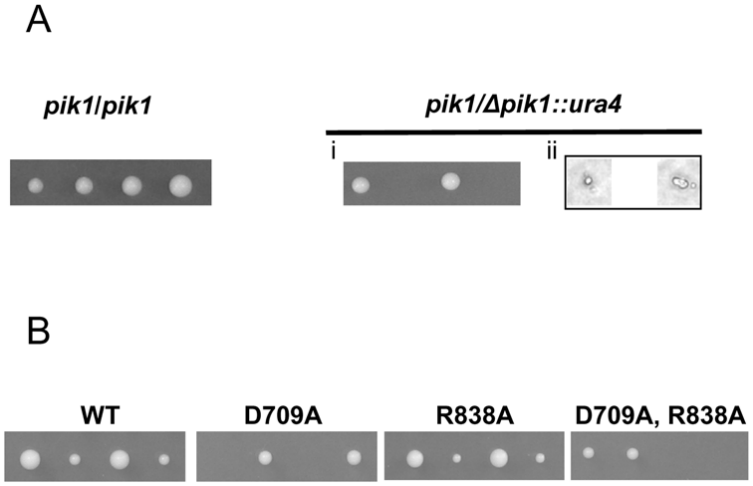
Tetrad analysis following *S. pombe pik1* disruption and mutant allele integration. (A) Diploid cells *pik1/pik1* or *pik1/Δpik1*::*ura4* (N1231) were incubated on ME plates to obtain azygotic asci. Spores were separated and incubated on YES plates. Results shown are representative of 8–10 asci. Each spore formed a colony when both chromosomal *pik1* loci were intact (left panel). Only two of the four spores formed colonies when only one chromosomal locus was intact (right panel; i). In cases where colonies did not form, the site of spore deposition was examined microscopically and photographed (right panel; ii). Many spores failed to germinate. Some spores germinated but the resulting cell divided only once. (B) A diploid strain (N1550) homozygous for the wild-type *pik1* coding region (WT), or heterozygous diploid strains carrying either the D709A substitution (N1565) or the R838A substitution (N1582), or both (N1596) were examined to evaluate the effects of the substitutions. For strains N1550 and N1582, each of four spores formed colonies. For strains N1565 and N1596, colonies were formed from only 2 of 4 spores. Results from one representative tetrad are shown for each strain.

Tetrad analysis was performed on the hemizygous diploid strain, *pik1/Δpik1*::*ura4*. Analysis of the germination and colony formation potential of spores from azygotic asci confirmed the essential nature of *pik1* at 30°C. Of the four spores from *pik1/Δpik1*::*ura4* diploid cells, only two formed colonies at 30°C (Figure 1A, right panel; i). These cells were wild-type, as determined by their growth requirement for uracil, and by the presence of *pik1* as determined by PCR analysis. Of the spores that did not form colonies, some remained spherical, most displayed some outgrowth and one germinated and divided once (Figure 1A, right panel; ii). In contrast, and as expected, the four spores from homozygous diploid strain *pik1/pik1* germinated and formed colonies at 30°C (Figure 1A). To evaluate if the requirement for *pik1* is affected by temperature, spores were incubated at 19°C, 25°C and 36°C. Only two of four spores formed colonies at these temperatures (not shown). Most of the spores that did not form colonies at 19°C or 25°C germinated and divided a few times, in contrast to most of the spores at 36°C which did not germinate or germinated, but did not divide. Thus, *pik1* is essential for vegetative cell division. *pik1* also appears to be required for spore germination at the higher temperatures (30°C–36°C).

Plasmid loss studies were carried out on a haploid *Δpik1:: Kan^R^* strain that carried an expressed episomal *pik1* cDNA sequence. To achieve this, the *pik1* coding region was replaced with a *Kan^R^* gene cassette in haploid cells that carried an episomal *pik1* cDNA sequence under the control of a highly attenuated (pREP81) thiamine-repressible *nmt1* promoter (strain N1113, Table 1). Cells were cultured without selection for the plasmid (+leucine). Repeated attempts to identify *Δpik1::Kan^R^* cells that had lost the episome failed. In addition, cells whose only source of *pik1* was from the episome, proliferated and had normal morphology when incubated in the presence or absence of thiamine (not shown). The *nmt1* promoter is leaky and some level of expression is observed, even in the presence of thiamine [51]. These results indicate that in cells carrying the *Δpik1*::*Kan^R^* chromosomal allele, the level of *pik1* expression from a thiamine-repressed, highly attenuated *nmt1* promoter is sufficient for cell growth and division.

### Pik1 is required for septation and cell separation

**Figure 2:**
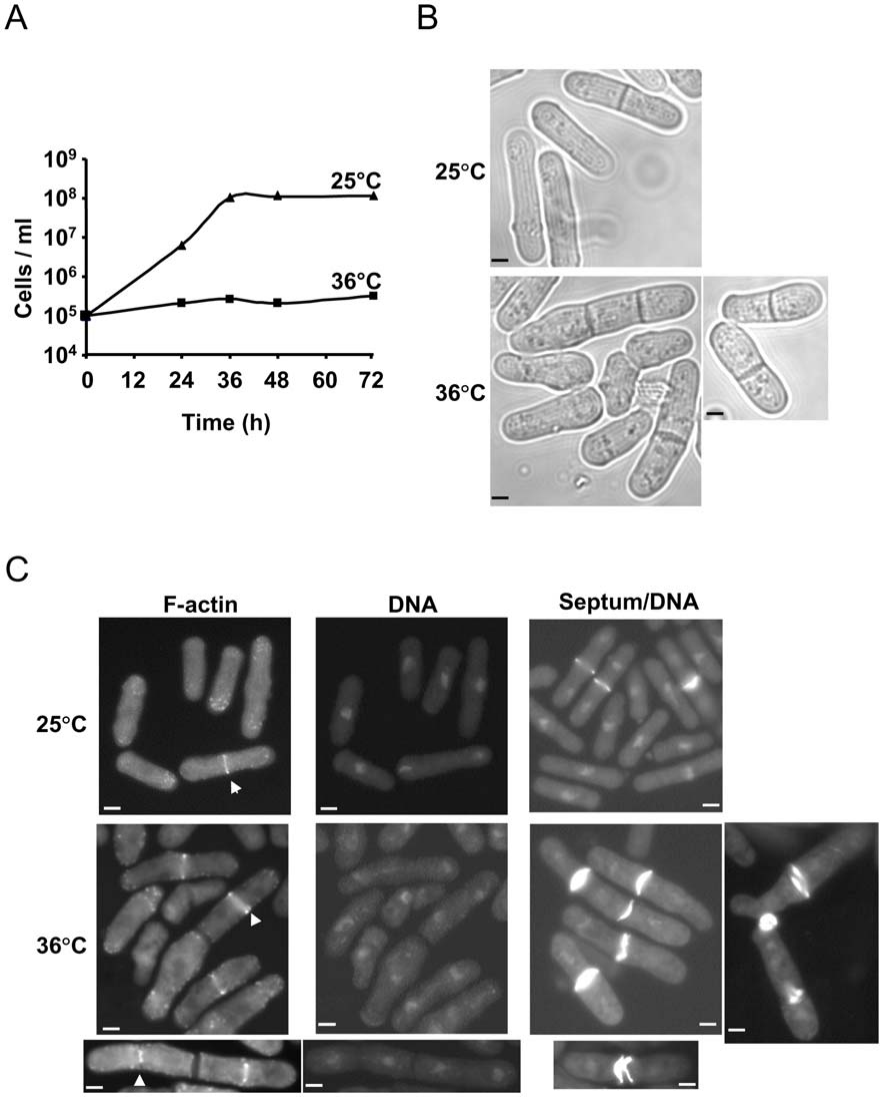
Cell proliferation and morphology of *pik1-td* cells at permissive and restrictive temperatures. *pik1-td* cells (N1366) carried *Δpik1::ura4* and pREP41X-Ub-R-DHFR^ts^-*pik1*. The plasmid expressed a thermolabile dihydrofolate reductase-Pik1 fusion protein. A shift from 25°C to 36°C is known to cause unfolding of the Ub-R-DHFR^ts^ fusion protein followed by ubiquitin dependent proteolysis (Dohmen *et al*., 1994). (A) At time 0, cultures at 25°C were shifted to 25°C (▴) or 36°C (▪) with thiamine present throughout. Cell proliferation ceased after a modest increase in cell number at 36°C. (B) Bright field microscopy of unfixed cells. Cells were incubated at 25°C for 24 h or incubated at 25°C for 12 h and then shifted to 36°C for 18 h. (C) Cells fixed with formaldehyde after 18 h at 25°C or 36°C were stained for F-actin (with FITC-phalloidin), DNA (DAPI) or septum (calcofluor white). Bars, 2 µm.

**Figure 3:**
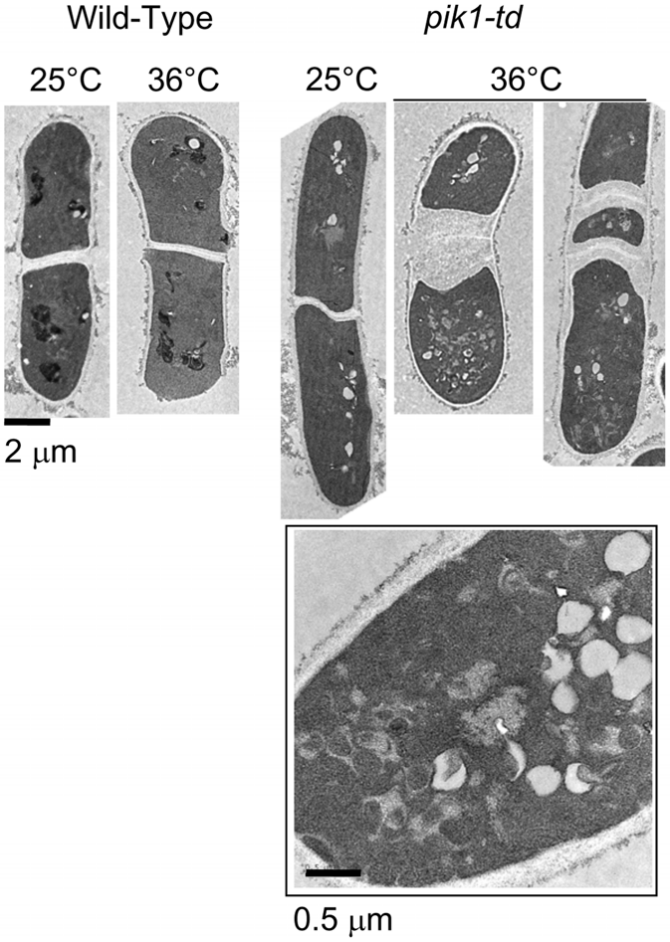
Septum morphology in *pik1-td* cells. Wild-type or *pik1-td* cells (N1366) were cultured for 18 h at 25°C or 36°C. Cells were fixed with potassium permanganate and processed for transmission electron microscopy. At 36°C, there was one or more septum per cell. The secondary septum were thickened relative to septum in *pik1-td* cells at 25°C or in wild-type cells at 25°C or 36°C. Intracellular membranous and vacuolar structures also accumulate in *pik1-td* cells at 36°C (magnified square).

To create a loss-of-function allele, the *pik1* cDNA coding region was fused to sequences encoding a ubiquitin, an arginine residue, and a temperature-sensitive dihydrofolate reductase fusion protein (Ub-R-DHFR^ts^) [40], [41]. This allele was designated *pik1-td* (*pik1*-temperature dependent). Ub-R-DHFR^ts^ is a thermolabile protein that unfolds at elevated temperatures to expose a destabilizing N-end residue, making the protein susceptible to degradation by ubiquitin-dependent proteolysis. At 25°C, the Ub-R-DHFR^ts^-Pik1 fusion protein should be stable. At 36°C, the Ub-R-DHFR^ts^ moiety should unfold and cause degradation of Pik1. The effects of loss of Pik1 function were assessed by ectopic expression of *pik1-td* under the control of an attenuated *nmt1* promoter in cells lacking the chromosomal *pik1* coding region (*Δpik1::ura4*). The *pik1-td* cells were cultured at 25°C in the presence of thiamine. These cells had a normal proliferation rate (division time = 3.6 h.; Figure 2A) and morphology (Figures 2B and C). F-actin was visualized at the cell tips and as a medial band. About 13–17% of the cells were binucleate and had an F-actin ring and a septum. Septa, visualized with calcofluor white (Figure 2C) or examined by transmission electron microscopy (Figure 3), had morphologies similar to wild-type cells. Thus, fusion of Ub-R-DHFR^ts^ to the N-terminal end of Pik1 had no apparent effect on Pik1 function at 25°C and it was sufficient for cell viability and proliferation. However, at 36°C, cell replication was suppressed with only a modest increase in cell number even after 72 h. in culture (Figure 2A). Within 18–20 h. after the temperature shift from 25°C to 36°C, cells appeared wider and many cells were elongated (Figure 2B). About 50% of the cells were binucleate and 15% had 3 or more nuclei. F-actin distribution was disrupted with F-actin patches dispersed throughout the cells rather than at one or both ends (Figure 2C). F-actin rings were observed in approximately 16% of the cells. Some cells had more than one F-actin ring. Apparent ring constriction was observed in some cells. Approximately 74% of the cell population had one or more septa (Figure 2C). About 33% of the cells seemed to be in the process of septum hydrolysis or to be arrested in the process of septation as they remained attached in a V-shape form through some residual septum (Figure 2B). Only about 6% of the cells showed this phenotype at 25°C (Figures 2B and C). The septum viewed by transmission electron microscopy is composed of three layers, one bright layer (primary septum) between two darker layers (secondary septa) [52]. Septum morphology of *pik1-td* cells incubated at 36°C was aberrant. These cells had one or more septa that were thickened, especially in the secondary septum layers, compared to the same cells at 25°C or wild type cells at either 25°C or 36°C (Figure 3). In addition, there was accumulation of intracellular membranous or vacuole-like structures in the *pik1-td* cells at 36°C (Figure 3). These structures were present to a much lesser extent at 25°C but were not observed in wild-type cells incubated at 25°C or 36°C. These observations indicate that temperature-dependent loss of Pik1 function arrests cells at a late stage in cytokinesis, preventing cell separation.

### Pik1, a Golgi associated protein, is also found at the medial region of dividing cells

**Figure 4:**
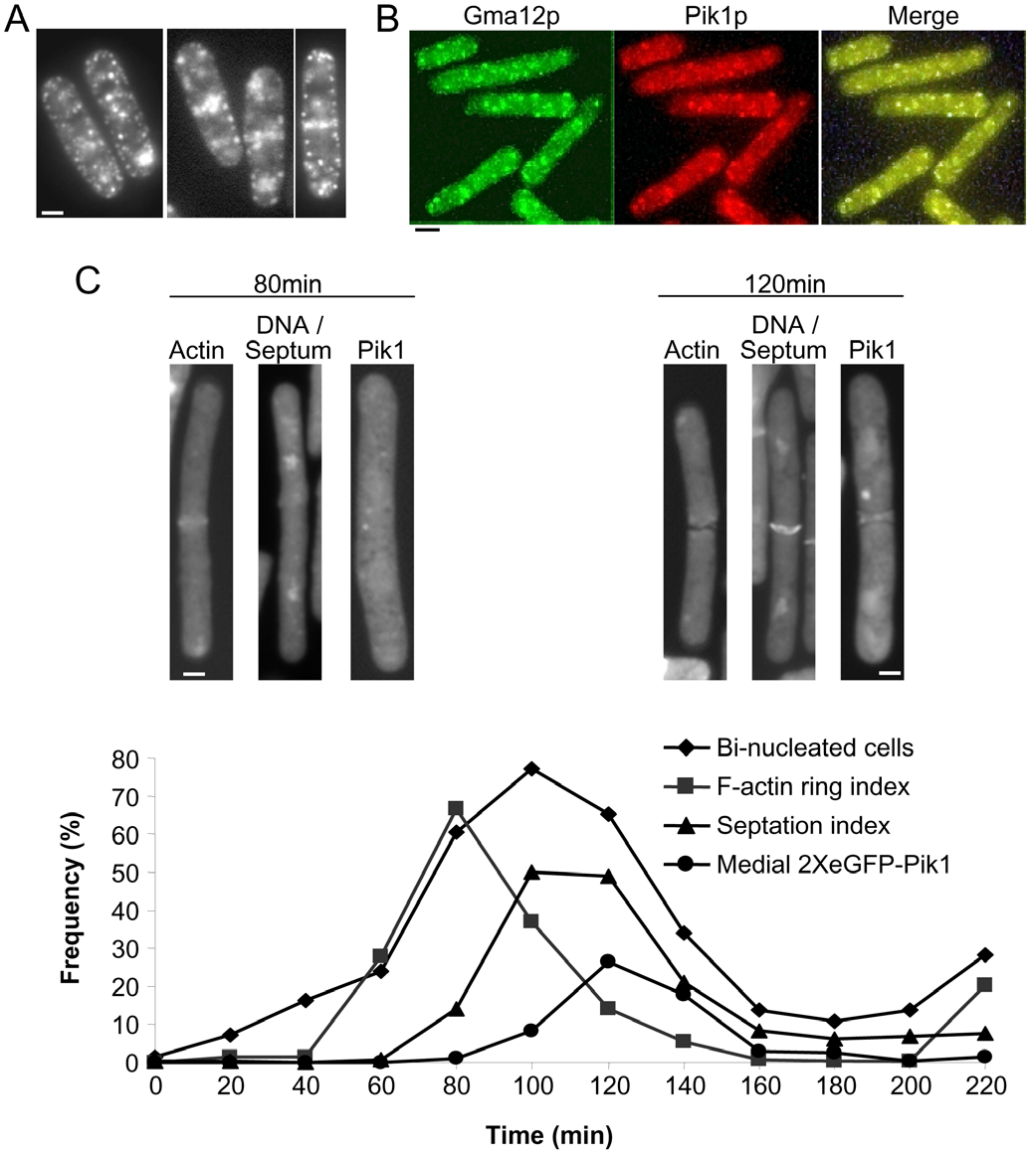
Localization of 2XeGFP-Pik1 in cells in asynchronous cultures, or in cultures synchronized by *cdc25-22* block and release. (A) N1369 cells carried *Δpik1::ura4* and pREP41-2XeGFP-*pik1*. The plasmid expressed 2 eGFP moieties, fused tandemly to the N-terminus of Pik1. Cells were cultured for 24 h at 30°C in the absence of thiamine. Punctate fluorescence was observed throughout the cytoplasm and around the periphery. A medial fluorescent band was observed in 8% of the cells. (B) Cells that carried a GFP-tagged allele of Gma12p (a Golgi-associated galactosyltransferase) were cultured for 24 hours at 30°C, fixed with methanol and processed for indirect immunofluorescence staining with rabbit antiserum against Pik1p and Texas Red conjugated, goat anti-rabbit antibodies. Cells were examined for Gma12p-GFP fusion (green) and Pik1p (red) and the images merged for colocalization (yellow). (C) N1401 cells which carried *cdc25-22* and pREP41-2XeGFP-*pik1*were synchronized by block and release. Cells were accumulated at G2/M by incubation at 36°C for 4 h and released to 25°C (time = 0 minute). Aliquots were taken every 20 minutes for microscopic examination for binucleate cells (nuclear DNA visualized with DAPI, ♦), F-actin ring index (visualized with FITC-phalloidin, ▪), septation index (visualized with calcofluor white, ▴) and 2XeGFP-Pik1 fluorescence at the medial region (•). Representative images are shown for cells collected 80 and 120 minutes after release from the temperature block. Results are representative of two independent experiments. Bars, 2 µm.

Sequences encoding two tandem eGFP (enhanced green fluorescent protein) proteins were fused to the 5′end of a *pik1* cDNA sequence in pREP41. Cells carrying this vector, pREP41-2XeGFP-*pik1*, and *Δpik1::ura4* were viable. The 2XeGFP-Pik1 protein fusion was stable, as western blots with anti-GFP and anti-Pik1 sera detected only the fusion protein in extracts from cells cultured in derepressed conditions (not shown). The fusion protein was below the detection limit in cells cultured under repressed conditions. Cells incubated in the presence or absence of thiamine had similar growth rates, morphology and septum and actin distributions (not shown). Thus, the fusion of 2XeGFP to Pik1 had no apparent effect on Pik1 functions. In the absence of thiamine, 2XeGFP-Pik1 fluorescence was observed as a punctate pattern throughout the cytoplasm and around the cell periphery. In about 8% of the cells, a fluorescent medial band was observed in addition to the dots (Figure 4A). No medial band or punctate fluorescence was observed within cells cultured under repressed conditions. The 2XeGFP-Pik1 punctate fluorescence was similar to that observed with indirect immunostaining with Pik1 antiserum. The latter co-localized with GFP-tagged Gma12p, a Golgi-associated galactosyltransferase (Figure 4B).

To evaluate the cell cycle dependence of recruitment of Pik1 to the medial region, 2XeGFP-*pik1* was expressed in *cdc25-22* cells synchronized by temperature block and release. At the restrictive temperature (36°C), *cdc25-22* cells arrest in G2/M [49]. After 4 h. at 36°C, cells shifted to 25°C enter mitosis and divide over the following 3 h. (Figure 4C). The mitotic index reached 80% in the synchronized cultures indicating a high level of induced synchrony. 2XeGFP-Pik1 first appeared at the medial region of the cells 100–120 min. after release. This corresponds approximately to the time of appearance of the septum (80–120 min.). Formation of the F-actin ring occured earlier (60–80 min.). The proportion of cells showing recruitment of 2XeGFP-Pik1 to the medial region peaked at only 30% at 120 min. after release. 2XeGFP-Pik1 fluorescence intensity may have been one limiting factor. Another possibility is that Pik1 recruitment to the medial region is poorly synchronized by the *cdc25-22* block and release method. The punctate fluorescence throughout the cytoplasm was still observed at all time points although it was quite faint. Thus, there is a cell cycle dependent recruitment of Pik1 to the medial region of dividing cells, at about the same time as deposition of septum material occurs.

### Pik1 residues D709 and R838 are required for lipid kinase and Cdc4-binding activities, respectively

**Figure 5:**
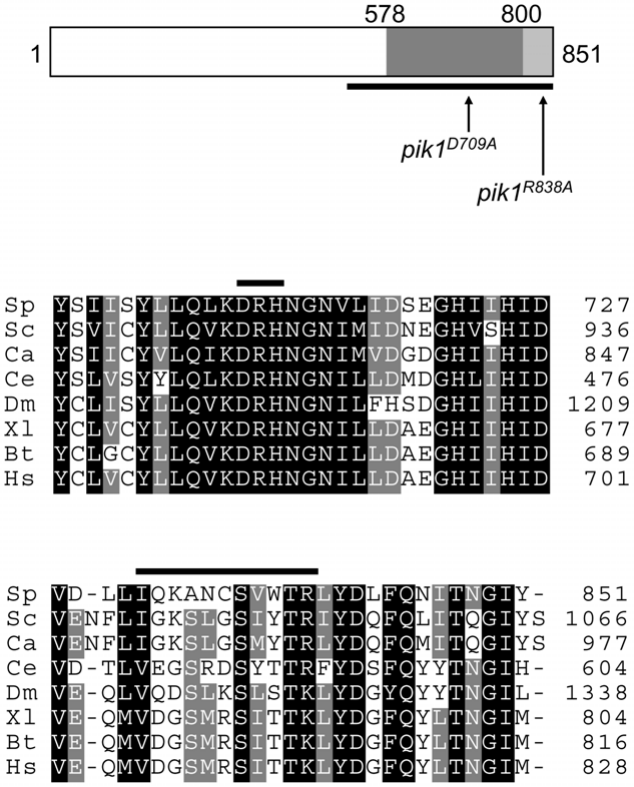
The C-terminal region of Pik1 is conserved. (A) Schematic representation of the primary structure of *S. pombe* Pik1 is shown. The kinase domain was identified from sequence similarity comparison to known lipid kinases using Prosite (grey shaded regions), or using Pfam (dark grey region only). A pseudo IQ motif is in the light grey shaded region. The positions of site-directed mutations in the kinase domain and in the pseudo IQ motif are indicated. (B) Sequence of the C-terminal region of *S.pombe* Pik1 (Sp; Accession CAA93903) is compared to those of orthologs in *Saccharomyces cerevisiae* (Sc; CAA53658), *Candida albicans* (Ca; CAA09718), *Caenorhabditis elegans* (Ce; NP_508177), *Drosophila melanogaster* (Dm; NP_728519), *Xenopus laevis* (Xl; Q6GN16), bovine (Bt; 002810) and human (Hs; BAA21661). The lipid kinase DRH motif and the C-terminal pseudo IQ motif (IQxxxRGxxxR) are indicated by black bands.

Previously, the 345 C-terminal amino acids of Pik1 (a.a. 507–851) were shown to interact with Cdc4 [21]. This region (Figure 5) includes the lipid kinase domain (a.a. 578–800) as identified by Pfam (http://pfam.sanger.ac.uk/) [53] or (a.a. 579–850) as identified by Prosite (http://ca.expasy.org/prosite/). To evaluate the importance of Pik1 lipid kinase and Cdc4-binding activities for cytokinesis, we created alleles that were impaired for each of these two functions.

A conserved aspartic acid residue, D709, within a DRH motif unique to lipid kinases, is one of several residues involved in ATP binding and phosphate transfer [54]. This residue was replaced with alanine to create *pik1^D709A^* (Figure 5). Equivalent mutations in other lipid kinases were shown to abolish kinase activity [54], [55], [56].

Cdc4 is known to bind to IQ domains of type II myosins [25], [26], [27]. A pseudo IQ motif (I_828_QKANCSVWTR_838_) is present in the C-terminal region of Pik1 (Figure 5). The first position of the IQ motif consensus sequence IQxxxRGxxxR is somewhat variable. It is generally Ile, Leu or Val, but Met, Phe and Thr are also found at this position in the IQ motifs of myosins [57]. In Pik1 in many species, the first position of the pseudo IQ motif is either Val or Ile (Figure 5). Furthermore, position 11 of the consensus sequence is typically occupied by an Arg or Lys, and this is also consistently observed within the pseudo IQ motifs of *pik1* homologs (Figure 5). The central residues of the IQ motif are less conserved [57]. To evaluate if this pseudo IQ motif was a binding site for Cdc4, a conserved arginine residue (R838) was replaced with alanine to create *pik1^R838A^* (Figure 5).

**Figure 6:**
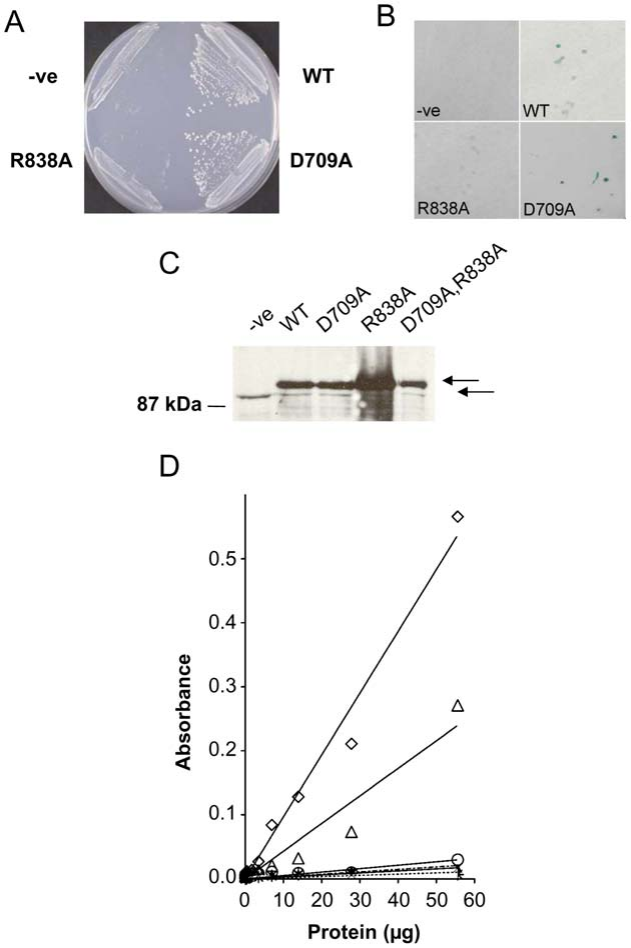
Pik1 R838, but not D709, is required for binding to Cdc4. (A) Yeast two-hybrid colony formation assay. *S. cerevisiae* cells were transformed with a two-hybrid bait vector carrying the *S. pombe cdc4* cDNA sequence fused to the *GAL4* DNA binding domain (Cdc4-*GAL4* DB). This strain was co-transformed with the two-hybrid prey vector alone (-ve), or with the prey vector carrying the *S.pombe pik1* C-terminal wild-type sequence fused to the *GAL4* transcription activation domain (Pik1^507–851^-*GAL4* TA; WT), or with the latter carrying either the D709A or R838A substitution. Colony formation is indicative of a protein-protein interaction. Results shown are representative of 3 separate experiments with the same strains. (B) X-gal colony filter assays. Nitrocellulose filters were overlayed onto the *S. cerevisiae* colonies grown on SD –Leu −Trp plates, submerged in liquid nitrogen and incubated in X-gal solution to monitor β-galacatosidase activity. Cells carrying the *pik1* wild-type and D709A alleles fused to the *GAL4*-TA domain in the pBI771 vector and *cdc4* sequence fused to the *GAL4*-BD in the pBI880 vector turned blue and were thus positive for the Pik1p-Cdc4p interaction. (C) Immunoblot assay. Accumulation of Pik1 upon ectopic expression of *pik1* alleles from an episome under the control of the *nmt1* promoter in cells carrying the intact chromosomal *pik1* locus was assessed by immunoblot analysis. *S.pombe* cells transformed with a pREP1 plasmid carrying either the full length *pik1* wild-type or mutated cDNA sequences were cultured in the absence of thiamine. Cell lysates (5 µg protein) were subjected to SDS-PAGE and Pik1 was visualized with a primary anti-Pik1 rabbit antiserum and a secondary goat anti-rabbit IgG-HRP (horseradish peroxidase)-conjugated antibody. Bar = 87 kDa molecular weight marker. Results shown are representative of 3 separate experiments with the same strains. The upper arrow represents the accumulation of the 97 kDa Pik1 wild-type and mutant proteins. The lower arrow represents an unknown polypeptide of 93 kDa, which is present in all samples including lysates of cells carrying the vector alone and lysates of cells cultured in repressed and derepressed conditions. (D) Sandwich ELISA to assess interaction between full length Pik1 and Cdc4. ELISA plates were coated with purified Cdc4, and then washed and blocked. Wells were subsequently incubated with 2-fold serial dilutions of lysates prepared from *S.pombe* cells grown in the absence of thiamine (solid line) and expressing the full-length *pik1* cDNA sequence (open triangle), or *pik1* cDNA sequences carrying mutations R838A (circle), D709A (diamond), or both R838A and D709A (cross hatch). Negative controls included lysates prepared from cells transformed with the pREP1 plasmid alone (dotted line, open square) or cultured under repressed conditions (with thiamine, dotted line, closed triangle). Results shown are representative of 3 separate experiments with the same strains.

A yeast two-hybrid assay, an ELISA and a lipid kinase assay were used to assess whether the R838A or D709A mutations affected binding of Pik1 to Cdc4, or Pik1 lipid kinase activity. For the yeast two-hybrid assay, *S. cerevisiae* cells were co-transformed with the ‘bait’ vector carrying the *S. pombe cdc4* cDNA sequence fused to the *GAL4*-BD (DNA binding domain) and with the ‘prey’ vectors carrying the C-terminal region of *S. pombe* wild-type and mutant *pik1* sequences fused to the *GAL4*-TA (transcriptional activation) domain. As observed previously [21], the C-terminal region of Pik1 interacted with Cdc4, as shown by colony formation on mimimal medium plates lacking leucine, tryptophan and histidine, and containing 3-AT (Figure 6A; WT). In addition, these cells possessed β-galactosidase activity, also a positive indicator of protein-protein interaction (Figure 6B; WT). Colony formation was not observed when cells were transformed with the ‘prey’ vector without the *pik1* sequence indicating that the *GAL4*-TA domain by itself did not result in a positive interaction phenotype (Figure 6A and B; -ve). When cells carrying the *GAL4*-TA *pik1^D709A^* fusion were examined for histidine prototrophy, colony formation was similar to that observed for cells carrying the *GAL4*-TA *pik1* wild-type allele (Figure 6A; D709A). These cells also possessed β-galactosidase activity (Figure 6B; D709A). In contrast, cells carrying the *GAL4*-TA *pik1^R838A^* fusion did not form colonies under these conditions, indicating that the Cdc4 interaction with Pik1 did not occur (Figure 6A; R838A). Doubly transformed cells carrying this *pik1* allele cultured in the presence of histidine formed colonies that were negative for β-galactosidase activity (Figure 6B; R838A).

As Cdc4 interaction with Pik1 in the yeast two-hybrid assay involved only the C-terminal end of Pik1, we wished to perform immunosorbent assays to assess the binding of Cdc4 to the full-length protein. Western blot analysis was performed to determine if the wild-type and mutant forms of Pik1 accumulated to detectable levels upon ectopic expression (Figure 6C). These cells also carried the *pik1* genomic locus. Lysates were prepared from cells carrying the ectopic *pik1* alleles and grown in repressed or derepressed conditions. Negative controls included cells carrying the vector alone without the *pik1* sequence. For each Pik1 allele, an equivalent amount of total protein was added and expression was analyzed under the same conditions. A band migrating at approximately 93 kDa was visible in all samples, including the negative control cells (Figure 6C, -ve). This band was also observed in lysates from negative control cells grown under repressed conditions (not shown). A band migrating at about 97 kDa, corresponding with the expected size of Pik1, was visible when any of the *pik1* alleles were expressed (Figure 6C). The signal after expression of the *pik1^R838A^* allele was reproducibly stronger than after expression of the other alleles. In lysates from cells grown under repressed conditions this band was either not visible or barely visible (not shown). Thus, derepression of the ectopic expression of each of the *pik1* alleles results in elevated levels of the corresponding protein in the cells.

For ELISA, wells were coated with purified Cdc4. Purified Cdc4 was obtained using a bacterial expression system and subsequent elutriation through an anion exchange column and gel filtration column [34]. One protein band was observed by SDS-PAGE and the protein identity was confirmed by mass spectrometry [34]. Subsequently the wells were incubated with serial dilutions of lysates of cells that carried *pik1* alleles cultured in the presence or absence of thiamine. Binding of Pik1 to Cdc4-coated wells was monitored with a primary antiserum against Pik1 and a secondary antibody-HRP conjugate (Figure 6D). Lysates from cells carrying the empty vector under derepressed conditions produced a very low ELISA signal (Figure 6D, open square). Since these cells carried the intact *pik1* locus this can be considered to be the background signal for this assay. Lysates from cells carrying the ectopic wild-type *pik1* allele and grown under repressed conditions, produced a background level ELISA signal (Figure 6D, closed triangle). Lysates from cells carrying the wild-type *pik1*allele and grown in the absence of thiamine, produced an increased Pik1 ELISA signal relative to that observed in the presence of thiamine (Figure 6D, open triangle). Pik1 with the D709A substitution also produced a positive ELISA signal that was reproducibly larger than that from Pik1 lacking this substitution (Figure 6D, open diamond). In contrast, Pik1 carrying the R838A substitution or carrying both the D709A and R838A substitutions, did not produce an ELISA signal that was above the background level. Thus, the Pik1^R838^ residue, which was shown to be required for the interaction with Cdc4 in the two-hybrid assay (Figure 6A) was also found to be required for interaction of the full length Pik1 protein with Cdc4-coated wells in ELISA (Figure 6D). We note that the abundance of the endogenous Pik1 protein is below or near the levels of detection in the ELISA and immunoblot assays.

**Figure 7:**
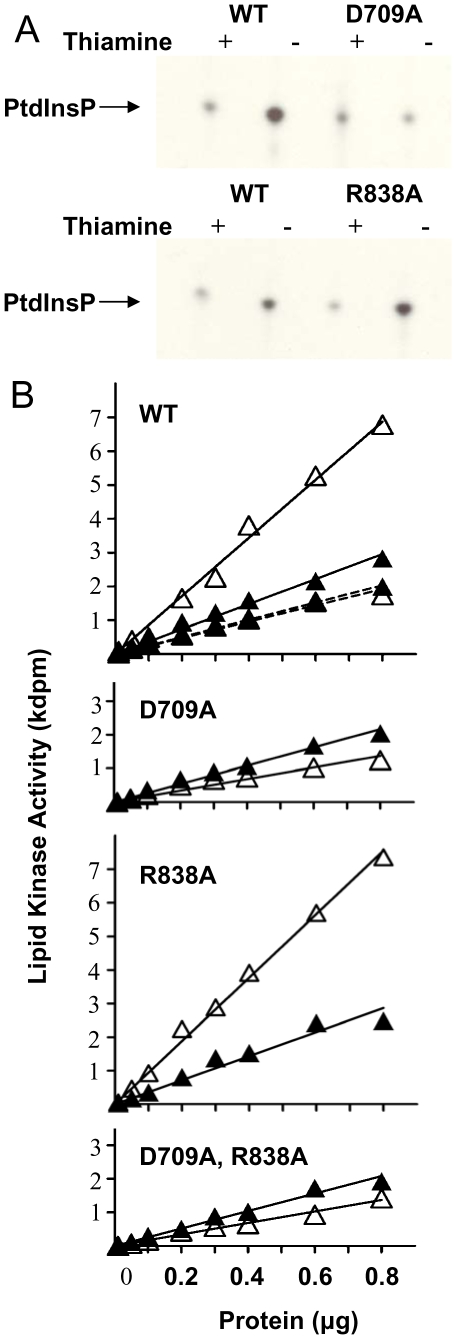
Pik1 D709, but not R838, is required for lipid kinase activity. (A) Incorporation of ^32^P into PtdInsP as visualized by autoradiography after chromatographic separation of phospholipids. *S. pombe* cells were transformed with pREP1 plasmids carrying either full-length wild-type (WT) or mutant (D709A or R838A) *pik1* cDNA coding regions under the control of the *nmt1* promoter. Cells were cultured in the presence or absence of thiamine as indicated. Cell lysates (0.05 µg total protein) were pulse labeled with [γ- ^32^P] ATP. Phospholipids were extracted and subjected to thin layer chromatography, followed by autoradiography. PtdInsP, monophosphorylated inositol phosphate. (B) Protein concentration dependency of lipid kinase activity in cell lysates. Lysates were prepared and assayed for lipid kinase activity as in (A). The lipid kinase activity in lysates prepared from cells carrying the empty pREP1 plasmid (WT, dotted lines), or the plasmid with the wild-type *pik1* sequence (WT, solid lines), incubated with thiamine (closed triangle) or without thiamine (open triangle) is shown in the top panel. Lipid kinase activity of lysates of cells after ectopic expression of *pik1^D709A^*, or *pik1^R838A^* or *pik1^D709A, R838A^* cultured under identical conditions are shown in the lower panels. Results shown are representative of 2 independent experiments using the same strains.

To evaluate the effects of the D709A and R838A substitutions on Pik1 enzymatic activity, lysates of cells expressing episomal *pik1* alleles were assayed for lipid kinase activity (Figure 7). These cells all carried the intact chromosomal *pik1* locus. Cells carrying each allele were grown for 24 hours at 30°C in repressed or derepressed conditions. Cell lysates containing a fixed amount of protein were pulse labeled with [γ- ^32^P] ATP and phospholipids were extracted and separated by TLC at the same time under the same conditions. The radiolabeled lipid spot in Figure 7A was identified as PtdInsP as it co-migrated with PtdIns(4)P but not with PtdIns(4,5)P_2_ or PtdIns(3,4,5)P_3_ reference lipid-standards (data not shown). PtdIns(4)P and PtdIns(3)P migrate with the same R_f_ value and are not resolved with this assay system [58], [59]. Lysates from cells carrying the wild-type *pik1* allele had higher incorporation of ^32^P label into PtdInsP when expression was derepressed (Figure 7A; WT, compare + and − thiamine). Lysates from cells carrying the *pik1^D709A^* allele and grown under repressed conditions showed incorporation of ^32^P label into PtdInsP that was similar to that of the wild-type allele under the same condition. Under derepressed conditions, incorporation appeared to be reduced (Figure 7A; D709A, - thiamine). In contrast, expression of the *pik1^R838A^* allele showed a level of induced lipid kinase activity that was comparable to or higher than that of the wild-type allele under the same conditions (Figure 7A; R838A).

Serial dilutions of cell lysates were also assayed for lipid kinase activity (Figure 7B). Lysates of cells transformed with the vector alone and incubated with or without thiamine showed a linear increase in lipid kinase activity over the range of protein tested (Figure 7B; WT, dotted lines). This activity represents endogenous activities, including those of the three putative PtdIns 4-kinases in *S. pombe*. In lysates of cells carrying the wild-type allele cultured in the presence of thiamine, an approximately 1.3-fold increase in lipid kinase activity was observed (Figure 7B; WT, solid line, closed triangle). Derepression of the wild-type allele resulted in an approximately 3-fold increase in lipid kinase activity (Figure 7B; WT, open triangle). These observations are consistent with the known behavior of the *nmt1* promoter under derepressed and repressed conditions [51]. Under repressed conditions, ectopic expression of *pik1^D709A^* resulted in lipid kinase activity that was comparable to that of the endogenous level (Figure 7B; D709A, closed triangle). When the expression of this allele was derepressed, the apparent lipid kinase activity was reproducibly reduced (Figure 7B; D709A, open triangle). It is likely that the Pik1^D709A^ protein that accumulated under these conditions competed with the endogenous enzymes for the phosphatidylinositol substrate, reducing the apparent lipid kinase activity. Ectopic expression of *pik1^R838A^* under repressed conditions resulted in lipid kinase activity that was similar to that observed with ectopic expression of the wild-type allele under the same condition (Figure 7B; R838A, closed triangle). Upon derepression, the activity increased approximately 3-fold (Figure 7B; R838A, open triangle). Lipid kinase activity in lysates of cells carrying *pik1^D709A, R838A^* was indistinguishable from that in lysates of *pik1^D709A^* cells (Figure 7B; D709A, R838A).

In conclusion, Pik1 with the D709A substitution has little or no lipid kinase activity, but retains its ability to interact with Cdc4. Pik1 with the R838A substitution retains lipid kinase activity, but does not bind Cdc4. Pik1 with both substitutions has little or no lipid kinase activity and does not bind Cdc4.

### Functional analysis of *pik1* alleles by ectopic expression

**Figure 8:**
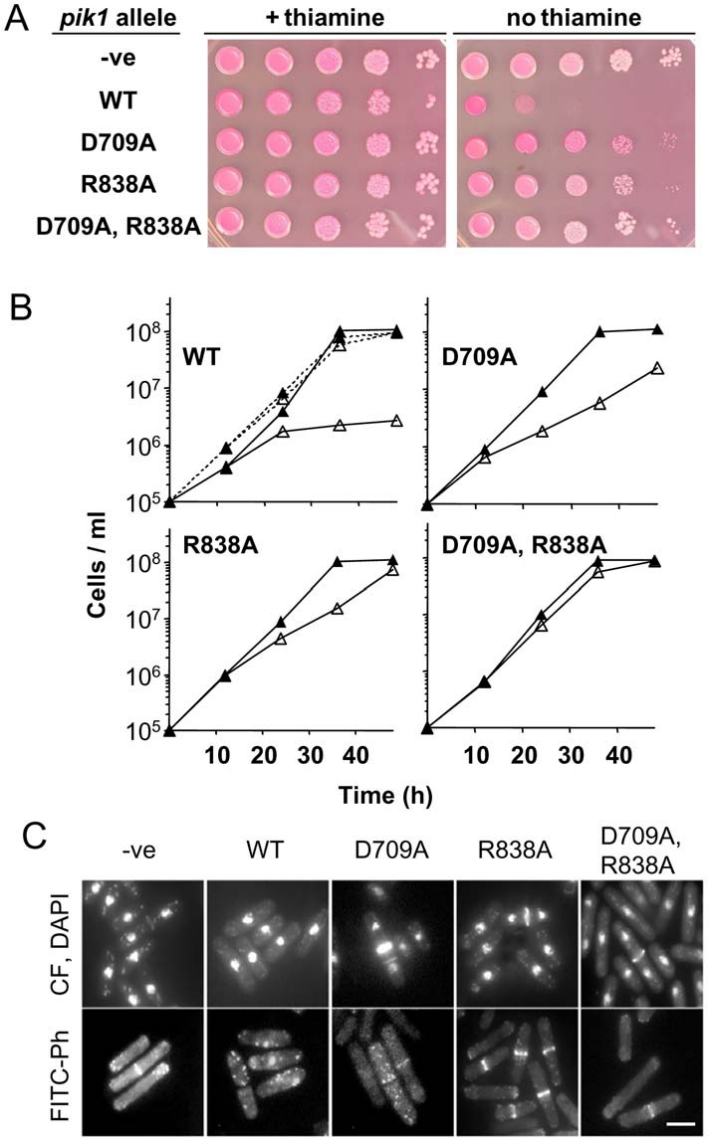
Effects of ectopic expression of *pik1* wild-type and mutant alleles on cell proliferation and morphology. (A) Colony formation assay. *S. pombe* cells transformed with the pREP1 plasmid with no insert (-ve), or with the pREP1 plasmid carrying *pik1* (WT), *pik1^D709A^* (D709A), *pik1^R838A^* (R838A), or *pik1^D709A, R838A^* (D709A, R838A) were cultured in liquid medium in the presence of thiamine to saturation. Cells were washed free of thiamine and serial 10-fold dilutions (about 10^5^ to 10^1^ cells per spot, from left to right) of each strain were spotted onto EMM –leucine plates with phloxin B and with (left panel) or without (right panel) thiamine. Plates were kept at 30°C for 6 days. (B) Cell proliferation in liquid culture. Cell cultures in EMM –leucine were started at a cell density of 1×10^5^ cells/mL and incubated at 30°C in the presence (closed triangle) or absence (open triangle) of thiamine. The dotted lines in the top left panel represent cells transformed with the pREP1 plasmid without the *pik1* cDNA sequence. Strain designations were as in (A). (C) Cell morphology. After 24 hours of growth in liquid culture (B), cells were fixed with formaldehyde and examined by epifluorescence microscopy after staining with calcofluor (to visualize the septum), DAPI (nucleus) or FITC-Phalloidin (FITC-Ph; F-actin). Bar = 5 µm. The results shown are for cell cultures in the absence of thiamine. Cell cultures in the presence of thiamine gave results similar to those observed with cells transformed with the vector alone (-ve). Results shown are representative of 2 (C) or 3 (A, B) separate experiments with the same cell strains. The relative frequency of binucleate cells and cells with F-actin rings or septa in the cell population are given in Table 3.

While performing the studies described above it was observed that ectopic expression of *pik1* was lethal under some circumstances. To investigate this further, the effects of ectopic expression of *pik1* alleles on colony formation and proliferation in liquid cultures were evaluated relative to cells carrying the vector alone (Figure 8A, B). Cells carrying the empty vector, or the wild-type or mutant alleles of *pik1* on an episome under the control of the full-strength *nmt1* promoter were serially diluted and spotted onto minimal medium plates with or without thiamine, and supplemented with the viability stain phloxin B. Phloxin B stains dead cells red [35]. After 6 days at 30°C in the presence of thiamine, cells carrying each of the *pik1* alleles grew indistinguishably from cells carrying the vector alone (Figure 8A, left panel). Similarly, the proliferation of these strains in liquid culture in the presence of thiamine were similar to each other and to the cells carrying the vector alone (Figure 8B). In the absence of thiamine, cells expressing the wild-type *pik1* sequence had a markedly reduced ability to form colonies, and the colonies were red (Figure 8A; WT, right panel). Proliferation of these cells after 24 hours in liquid culture in the absence of thiamine, was markedly reduced (Figure 8B; WT, solid line, open triangle) compared to cell cultures with thiamine (Figure 8B;WT, solid line, closed triangle) or to cells carrying the vector alone in repressed or derepressed conditions (Figure 8B; dotted lines, open and closed triangles). Cells expressing *pik1^D709A^* formed colonies, but the colonies were red indicative of some cell death (Figure 8A; D709A, right panel). In liquid culture in the absence of thiamine, these cells showed reduced proliferation (Figure 8B; D709A, open triangle). Colony formation of cells expressing *pik1^R838A^* was similar to that of cells carrying the vector alone (Figure 8A; R838A, right panel). These cells in liquid culture in the absence of thiamine had a slightly reduced rate of proliferation (Figure 8B; R838A, open triangle). Colony formation of cells ectopically expressing the double-mutant *pik1^D709A, R838A^* was also similar to that of cells carrying the vector alone (Figure 8A; D709A, R838A, right panel). The proliferation of these cells in liquid cultures was very similar under repressed and derepressed conditions and to cells carrying the vector alone (Figure 8B; D709A, R838A, closed and open triangle).

**Table 3:** Effects of ectopic expression of *pik1* alleles on cytokinesis in *cdc4* cells.

*pik1* allele	+ thiamine					− thiamine				
	Length	CR-A	CR-M	Septa	BiN	Length	CR-A	CR-M	Septa	BiN
	(µm)	(frequency, %)				(µm)	(frequency, %)			
-	13.4±0.2	8	10	9	6	13.4±0.3	10	9	9	9
WT	13.6±0.2	9	9	9	10	11.5±0.4	1	1	10	10
D709A	13.8±0.2	8	9	10	11	15.6±0.4	8	8	20	29
R838A	13.3±0.2	14	8	9	10	12.4±0.2	14	8	10	13
D709A, R838A	13.1±0.2	8	8	8	10	13.6±0.3	10	8	7	10

Cells were cultured at 30°C for 24 hours, + or − thiamine. Cell length at division (cells with septa) was measured and expressed as mean±S.E., n = 25–50 cells. Contractile ring indices were estimated as the proportion of the cells with medial bands containing F-actin (CR-A) or myosin (CR-M), as visualized with FITC-phalloidin or indirect immunofluorescence, respectively. Similarly, septum indices were estimated with calcofluor-white (Septa), and the frequencies of binucleate cells with DAPI (BiN). n = 100–450 cells.

Thus, ectopic expression of the wild-type *pik1* allele in cells carrying the intact *pik1* chromosomal locus was lethal. The Pik1 activities that require the R838 residue accounted for most of the lethal effect of ectopic expression of *pik1*, while the activities that require the D709 residue contributed to a lesser extent.

### Morphological analysis of cells after ectopic expression of *pik1* alleles

In liquid cultures after 24 hours at 30°C, with or without thiamine, about 10% of cells carrying the vector alone showed actin and myosin medial bands, were binucleate and had normal septa (Figure 8C; -ve; Table 3). Cells ectopically expressing the wild-type *pik1* allele had reduced cell length at division relative to cells cultured in repressed conditions, or to cells carrying the vector alone (Table 3). About 10% of the cell population had 2 nuclei and a septum, a frequency similar to that of cells carrying the vector alone or cultured under repressed conditions (Table 3). However, septum morphology was abnormal in some cells as defined by more intense calcofluor staining and/or of aberrant shape (Figure 8C; WT). Remarkably, only about 1% of the cell population ectopically expressing the wild-type *pik1* allele formed actin and myosin rings (Table 3). Actin cytoskeletal structures visualized with FITC-phalloidin were disrupted with punctate actin staining evident throughout the cell instead of at the cell poles or medial region as observed in cells grown in the presence of thiamine or carrying the vector alone (compare Figure 8C; WT with Figure 8C; -ve). Thus, ectopic expression of the wild-type *pik1* sequence, resulting in the accumulation of an active lipid kinase (Figure 7), inhibits cell proliferation (Figure 8), presumably by disrupting actin cytoskeletal and septum structures.

The most obvious effect of ectopic expression of *pik1^D709A^* was a marked increase in septation index as approximately 20% of the cell population had 2 nuclei and a septum (Table 3). The mean cell length at septation was also longer relative to cells grown under repressed conditions or carrying the vector alone (Table 3). In a third of these cells, calcofluor white staining showed intense fluorescence. Some cells had two or more septa (Figure 8C; D709A). The number of cells with actin or myosin rings was similar to that for *pik1^D709A^* cells grown in repressed conditions or cells carrying the vector alone (Table 3). Staining with FITC-phalloidin revealed some cells with disrupted F-actin structures (Figure 8C; D709A). These results suggest that expressing a *pik1* allele with no lipid kinase activity (Figure 7), interferes primarily with septation, and may have some effects on F-actin structures although contractile ring formation appeared to be unaffected.

Under derepressed conditions, cells carrying the *pik1^R838A^* allele had slightly reduced cell length at septation relative to cells cultured in repressed conditions or to cells with the vector alone (Table 3). The number of cells with actin rings, myosin rings and septa were similar to cells cultured in repressed conditions and to cells carrying the vector alone (Table 3 and Figure 8C). Similarly, septum appearance was unaffected (Figure 8C; R838A). Thus, the disruption of actin-myosin structures and the abnormal septum morphologies observed with the ectopic expression of the wild-type *pik1* sequence were not observed when the R838 residue was replaced with alanine.

Cells ectopically expressing *pik1^D709A, R838A^* were morphologically wild-type (Figure 8C, Table 3). These results emphasize the importance of both the D709 and R838 residues in the functions of Pik1.

### The *pik1* ectopic expression lethal phenotype is suppressed in *cdc4^G107S^* cells

**Figure 9:**
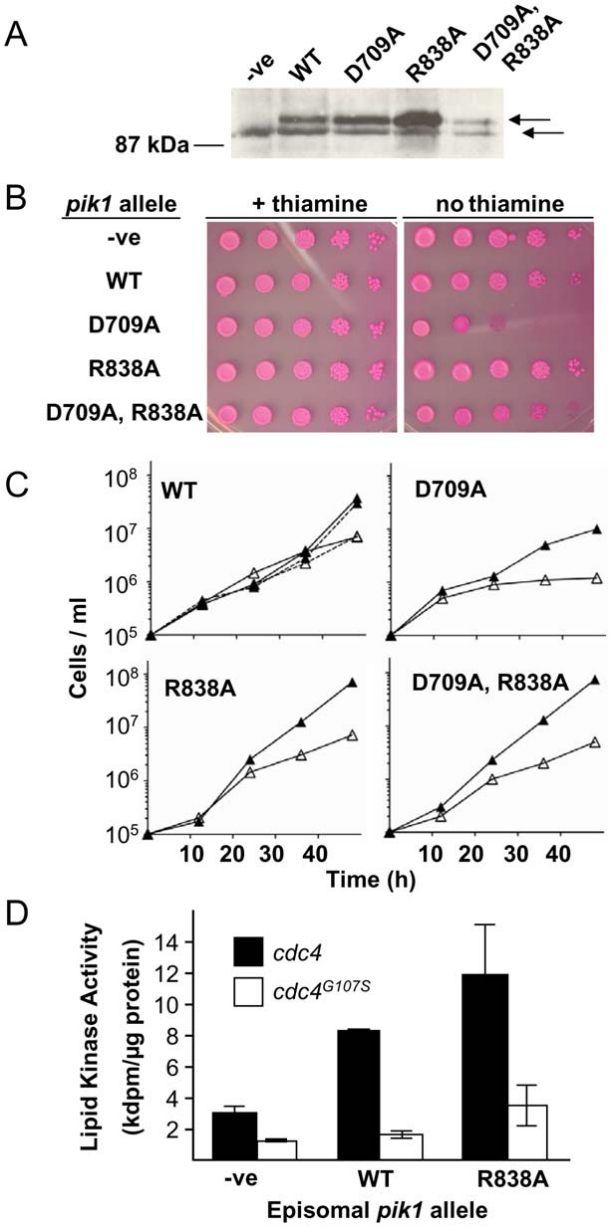
Effects of ectopic expression of *pik1* alleles in *cdc4^G107S^* cells. (A) Immunoblot assay. Accumulation of Pik1 in *cdc4^G107S^* cells upon ectopic expression of *pik1* alleles from an episome under the control of the *nmt1* promoter in cells carrying the intact chromosomal *pik1* locus was assessed by immunoblot analysis as described for Figure 6C. Results shown are representative of 3 separate experiments with the same strains. Colony formation assays (B) and proliferation in liquid medium (C) of *S. pombe cdc4^G107S^* cells transformed with the pREP1 plasmid with no insert (-ve), or with the pREP1 plasmid carrying *pik1* (WT), *pik1^D709A^* (D709A), *pik1^R838A^* (R838A), or *pik1^D709A, R838A^* (D709A, R838A) were carried out as described in Figure 8A and B. (D) Lipid kinase activity assays. Cells carrying either *cdc4* or *cdc4^G107S^* were transformed with a pREP1 plasmid with no insert (-ve), or with wild-type *pik1^wt^* (WT) or *pik1^R838A^* (R838A) cDNA sequences. Cells were incubated at 25°C for 24 hours in the absence of thiamine, and lysates prepared and assayed immediately for lipid kinase activity as described in Figure 7. Results shown are mean±S.E. of at least 3 separate experiments with the same strains.

The R838 residue of Pik1 is required to bind Cdc4 (Figure 6) and for *pik1* ectopic lethality (Figure 8). We reported previously that one mutation in *cdc4*, the G107S substitution, prevented the interaction of Pik1 with Cdc4 [21]. We hypothesized that ectopic expression of wild-type *pik1* might not be lethal in cells carrying the *cdc4^G107S^* allele, just as ectopic expression of *pik1^R838A^* had little effect in wild-type cells (Figure 8; Table 3). Ectopic expression of each of the *pik1* alleles in *cdc4^G107S^* cells after 24 hours at 30°C resulted in accumulation of the corresponding protein as determined by immunoblotting (Figure 9A) and was similar to that observed when these alleles were expressed in *cdc4* cells (Figure 6C). Of note, as in *cdc4* cells, Pik1^R838A^ reproducibly accumulated in *cdc4^G107S^* cells to higher levels than the other Pik1 variants (Figure 9A; R838A) and Pik1^D709A, R838A^ abundance appeared to be slightly reduced (Figure 9A; D709A, R838A).

In colony formation assays at 30°C in the presence of thiamine, *cdc4^G107S^* cells carrying each of the episomal *pik1* alleles formed colonies indistinguishably from *cdc4^G107S^* cells carrying the empty vector (Figure 9B, left panel). In the absence of thiamine, cells ectopically expressing the wild-type *pik1* sequence also formed colonies similarly to cells carrying the empty vector (Figure 9B; WT, right panel). This was in marked contrast to the lethal effect of *pik1* ectopic expression in cells carrying the wild-type *cdc4* allele (compare WT in Figure 8A and 9B, right panels). Expression of *pik1^D709A^* markedly reduced the ability of *cdc4^G107S^* cells to form colonies (Figure 9A; D709A, right panel), also in contrast to the expression of the same allele in wild-type *cdc4* cells (Figure 8A, right panel). Colony formation of *cdc4^G107S^* cells expressing *pik1^R838A^* was similar to that of cells carrying the empty vector (Figure 9B; R838A, right panel). Expression of *pik1^D709A, R838A^* had some effect on colony formation in *cdc4^G107S^* cells, intermediate between the growth suppression seen with expression of *pik1^D709A^* and the apparent viability of cells expressing *pik1^R838A^* (Figure 9B; D709A, R838A, right panel). The same results were observed when the cells were grown at 25°C (not shown).

Cell proliferation by these strains in liquid cultures at 25°C was consistent with the results of the colony formation assays (Figure 9C). Namely, *cdc4^G107S^* cells ectopically expressing wild-type *pik1* grew similarly to cells incubated in the presence of thiamine and to cells carrying the empty vector (Figure 9C; WT). Ectopic expression of each of the mutant alleles reduced cell proliferation to some extent compared to proliferation of cells ectopically expressing the wild-type *pik1* allele (Figure 9C). In both the colony formation assay and in liquid culture the greatest effect was from ectopic expression of *pik1^D709A^* (Figures 9B and 9C).

Morphological analysis was performed on *cdc4^G107S^* cells after ectopic expression of each of the *pik1* alleles (Table 4). Expression of the wild-type *pik1* allele resulted in a slight reduction in cell length at septation, but no effects were observed on the frequencies of cells with F-actin rings, with two nuclei or with septa (Table 4). This was in contrast to the marked reduction of the F-actin ring index in *cdc4* cells after ectopic expression of wild-type *pik1* (Table 3). The suppression of the ectopic *pik1* phenotype in the *cdc4^G107S^* strain was allele specific since it was not observed in the *cdc4^F12L^*, *cdc4^G19E^*, *cdc4^R33K^* and *cdc4^G82D^* backgrounds (not shown). Cells ectopically expressing the *pik1^D709A^* allele had cell lengths at septation, and septum and contractile ring formation comparable to cells cultured under repressed conditions or carrying the empty vector (Table 4). Interestingly, expression of this allele had the greatest negative effect on cell proliferation and colony formation. The most pronounced morphological effect observed was in cells in which the *pik1^R838A^* allele was ectopically expressed. Compared to cells carrying the empty vector with or without thiamine, under repressed conditions these cells were 10% shorter at septation, and under derepressed conditions, 25% shorter (Table 4). Notably, the proliferation and colony formation of these cells appeared to be normal. Cells in which *pik1^D709A, R838A^* was ectopically expressed were slightly longer at septation and had increased binucleate and septum indices (Table 4). The number of these cells with F-actin staining at the medial region was similar to cells carrying the vector alone or cultured in repressed conditions.

**Table 4:** Effects of ectopic expression of *pik1* alleles on cytokinesis in *cdc4* ^G107S^ cells.

*pik1* allele	+ thiamine					− thiamine				
	Length	CR-A	CR-M	Septa	BiN	Length	CR-A	CR-M	Septa	BiN
	(µm)	(frequency, %)				(µm)	(frequency, %)			
-	10.4±0.5	11	-	18	20	10.9±0.5	11	-	18	20
WT	10.4±0.4	10	-	17	23	9.2±0.2	10	-	16	18
D709A	9.8±0.4	9	-	17	23	10.6±0.4	15	-	20	29
R838A	9.3±0.3	9	-	15	19	8.1±0.2	9	-	25	24
D709A, R838A	10.8±0.4	10	-	15	23	12.6±0.7	8	-	26	35

Experiments was performed as described for Table 3, except cells were cultured at 25°C and indirect immunofluorescence for myosin was not done.

Overall, ectopic expression of wild-type *pik1* in *cdc4^G107S^* cells had little to no effect on cell proliferation, in marked contrast to the lethality observed in *cdc4* cells. In *cdc4^G107S^* cells, it was ectopic expression of the kinase dead allele (*pik1^D709A^*) that caused dominant lethality (Figures 9B and C).

Since the lethality caused by the ectopic expression of wild-type *pik1* was not observed in *cdc4^G107S^* cells, we evaluated if the lipid kinase activity of Pik1 was altered in this background. Cells ectopically expressing wild-type *pik1* or *pik1^R838A^* were incubated at 25°C for 24 h. In accord with preceding results (Figure 7B), ectopic expression of either wild-type *pik1* or *pik1^R838A^* in *cdc4* cells resulted in a 3 to 4-fold increase in lipid kinase activity in the cell lysates relative to lysates of cells carrying the empty vector (Figure 9D; black bars). In contrast, this was not observed with expression of the same alleles in *cdc4^G107S^* cells (Figure 9D; WT, open bar). Some increase in cell lysate lipid kinase activity may have been observed with ectopic expression of *pik1^R838A^* (Figure 9D; R838A, open bar). Thus, although both wild-type Pik1 and Pik1^R838A^ accumulated in *cdc4^G107S^* cells (Figure 9A), a corresponding increase in lipid kinase activity was not observed (Figure 9D).

### Pik1 lipid kinase activity is essential

We used two approaches to determine if Pik1^D709^ and Pik1^R838^ have essential functions. First, we determined the phenotypes of cells carrying these substitutions in the *pik1* chromosomal locus. Second, we determined if *S. pombe* wild-type and mutant *pik1* alleles could complement the conditional lethality of an *S. cerevisiae* strain that carried *pik1-101*, an allele that has been shown to have little or no lipid kinase activity at the restrictive temperature [7].

Heterozygous diploid strains (Table 1; N1550, N1565, N1582, N1596) were constructed in which one copy of the *pik1* locus was fully wild-type, while the other was either wild-type with respect to the coding sequence or carried either the R838A or D709A substitutions, or both. We note that the modified loci also had the *pik1* 3′-untranslated region displaced by the *nmt1* terminator sequence and a *ura4* cassette (*pik1::term^nmt1^::ura4*). These diploid strains were incubated on ME plates at 25°C for 2 days to obtain azygotic asci which were transferred to YES plates. Spores from each of 8–10 asci were separated with a micromanipulator and incubated at 30°C for 5 days. As a control, strain N1550 was constructed in which one copy of the *pik1* locus was fully wild-type, while the other copy was wild-type with respect to the coding sequence, but had the same modifications to the 3′-untranslated region. In 8 of 10 tetrads, each of the 4 spores formed a colony, although 2 colonies were much larger (Figure 1B; WT). The larger colonies grew on plates lacking uracil indicating that they contained the 3′ end modifications (not shown). Uracil prototrophy may confer a growth advantage. Analysis of the germination and growth potential of spores from 8 azygotic asci from the *pik1/pik1^D709A^* strain (N1565) revealed that the D709 residue is essential, presumably reflecting a requirement for Pik1 lipid kinase activity at 30°C. In 6 of 8 azygotic asci dissected, only 2 of the 4 spores formed colonies (Figure 1B; D709A). The cells of these colonies were wild-type for *pik1*, as confirmed by sequencing and their growth requirement for uracil. Microscopic examination of the spores that did not form colonies revealed that the majority had germinated, the cells elongated, but failed to divide (not shown). In contrast, four colonies were observed from each tetrad in 8 of 10 azygotic asci dissected from the *pik1/pik1^R838A^* strain (N1582) (Figure 1B; R838A), indicating that *pik1* functions affected by this mutation are not essential. In 7 of 9 azygotic asci dissected from the *pik1/pik1^D709, R838A^* strain (N1596), only 2 of the 4 spores formed colonies and these cells were wild-type for *pik1* (Figure 1B; D709A, R838A). Variable phenotypes were observed upon microscopic examination of the spores that failed to form colonies. Some spores failed to germinate, another grew into one cell that failed to divide while others grew into cells that divided a few times (not shown). The functions affected by the R838A mutation are dispensable for vegetative growth while Pik1 lipid kinase activity appears to be essential (Figure 1B).

**Figure 10:**
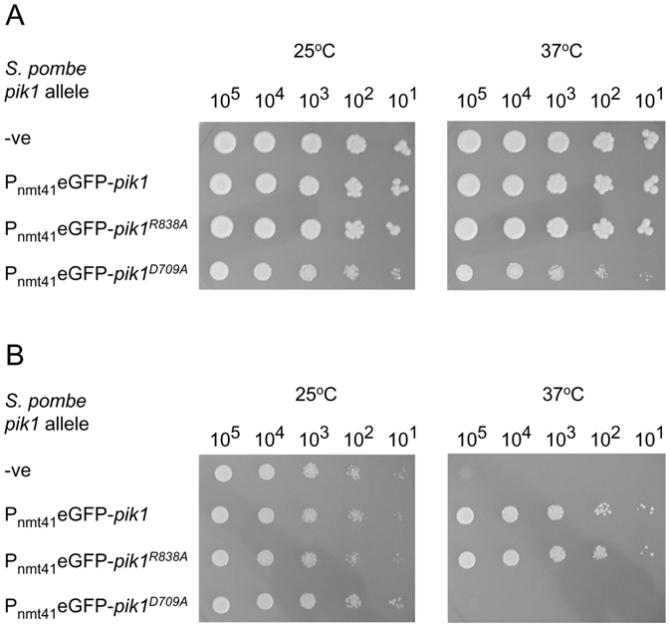
Complementation of *S.cerevisiae pik1-101* by heterologous expression of *S.pombe pik1* alleles. Colony formation assays. (A) *S.cerevisiae PIK1* cells were transformed with empty YEplac181 as negative control (-ve), or with YEplac181 recombinants that expressed eGFP fusions to wild-type Pik1 under the control of an attenuated *nmt1* promoter (P*_nmt41_* -eGFP-*pik1*), or the same construct carrying either the D709A or R838A substitutions. To assay for colony formation at 25°C or 37°C, aliquots from serial dilutions of each culture, containing the number of cells indicated, were prepared and spotted onto SD-Leu plates lacking thiamine which were incubated for 5 days. (B) The experiment as described in (A) was performed with *S.cerevisiae pik1-101* cells. The colony formation assays were replicated independently at least three times. The results shown are representative of each of the replicates.

We wished to perform complementation studies to determine if the essential functions of *S. cerevisiae PIK1*, which are lost in *pik1-101* cells at the restrictive temperature, can be provided by expression of the *S. pombe pik1* gene. However, *S. pombe* cells are highly sensitive to changes in *pik1* expression (Figure 8). If *S. cerevisiae* cells are similarly sensitive to changes in *PIK1* expression, and if *S. pombe* Pik1 is active and capable of providing the essential functions of Pik1 in *S. cerevisiae*, then improperly regulated expression of *pik1* might result in lethality, thus confounding the experimental approach. Searching for a method to achieve a range of levels of *S. pombe pik1* expression in *S. cerevisiae*, we hypothesized that the thiamine-repressible *S. pombe nmt1* promoter might function in *S. cerevisiae* and provide such a method. We therefore placed *S. pombe pik1* cDNA sequences into the *S. cerevisiae* plasmid, YEplac181 [49], under the control of either the wild-type *nmt1* promoter (P*_nmt1_*), or an attenuated (P*_nmt41_*) or a highly attenuated (P*_nmt81_*) version of the promoter [38]. The *nmt1* terminator sequence was included in these expression cassettes. We performed all of the experiments described below both in the presence and absence of thiamine. Since the results were indistinguishable only those experiments in the absence of thiamine are shown (Figure 10). In initial studies with the wild-type *pik1* coding sequence, we reproducibly observed a slight reduction in colony formation efficiency at 37°C in *PIK1* cells in which P*_nmt1_* controlled *S. pombe pik1* expression (not shown). This effect was not observed when the attenuated promoters were used. We observed reproducible, but partial complementation of the lethal phenotype of *pik1-101* at 37°C with P*_nmt1_*, reduced partial complementation with P*_nmt41_*, and no complementation with P*_nmt81_* (not shown). These results indicate that *S. pombe pik1* can provide essential functions of Pik1 in an *S. cerevisiae* loss-of-function mutant and that *S. pombe nmt1* promoter sequences are useful in *S. cerevisiae*. Immunoblot analysis, using a polyclonal anti-*S. pombe* Pik1 serum that detected this protein in *S. pombe* cell extracts (Figure 6C), failed to detect it in extracts from transformed *S. cerevisiae pik1-101* cells (not shown).

To study the subcellular distribution of *S. pombe* Pik1 in *S. cerevisiae*, we expressed an eGFP-*pik1* fusion allele under the control of P*_nmt41_* in *S. cerevisiae PIK1* and *pik1-101* cells. Unfortunately, the fluorescent signal from the eGFP-*S. pombe* Pik1 fusion protein was insufficient for the purpose of determining its subcellular distribution. Immunoblot analysis again failed to detect this protein in extracts from transformed *S. cerevisiae PIK1* cells (not shown). Colony formation assays revealed that expression of eGFP-*pik1* in *PIK1* cells was innocuous at both 25°C and 37°C (Figure 10A), as it was in *pik1-101* cells at 25°C (Figure 10B). Remarkably, eGFP-*pik1* fully complemented the lethality of *pik1-101* at 37°C (Figure 10B). We then introduced the D709A and R838A substitutions into this construct. Expression of eGFP-*pik1^D709A^* in *PIK1* cells appeared to impair colony formation at both temperatures assayed as compared to the control cells that lacked the *pik1* sequences or the cells expressing the eGFP-*pik1* allele (Figure 10A). This allele failed to complement *pik1-101* at the restrictive temperature (Figure 10B). In contrast, eGFP-*pik1^R838A^* had no apparent deleterious effect in *PIK1* cells (Figure 10A) and it fully complemented *pik1-101* at the restrictive temperature (Figure 10B).

## Discussion

### Pik1 localizes to the medial region of dividing cells and is required for cell division

*S. pombe* cells divide by medial fission. The contractile ring assembles at the onset of mitosis [24], [60]. A multilayered septum forms in concert with constriction of the ring. Cleavage of the primary septum, the inner layer, results in cell separation [61]. The exocyst is a multiprotein complex present in many cells and which appears to be involved in the recruitment of Golgi vesicles to the division site, using positioning cues provided by the contractile ring [45], [62]. In *S. pombe*, the exocyst is involved in the recruitment of enzymes responsible for septum cleavage and is essential for cell separation [63]. *S. pombe* Pik1 is associated with the Golgi [18]. We have shown here that *pik1* is an essential gene, like its *S. cerevisiae* orthologue [12]. Haploid *Δpik1::ura4* cells failed to divide (Figure 1), and *pik1::ura4* cells carrying the temperature-sensitive *pik1-td* fusion stopped dividing when shifted to the restrictive temperature (Figure 2). These cells accumulated intracellular membranous and vacuolar materials (Figure 3). Similar structures were observed in the absence of the *S. cerevisiae PIK1* functions and were identified as abnormal Golgi structures [7], [13]. Likewise, expression of a kinase-dead allele of a mammalian *pik1* homolog resulted in disrupted Golgi structures in mammalian cells [64]. Thus, some Pik1-dependent Golgi function is likely required for cell division in *S. pombe*. These results also suggest that the essential functions of Pik1 are not redundant to the functions of the other two putative *S. pombe* PtdIns 4-kinases.

*S. pombe* Pik1 was also found in the medial region of dividing cells at the time of septum formation (Figure 4). The Pik1 medial localization has not been reported previously [18], possibly because of low abundance of Pik1. Alternatively, fusion of GFP to the C-terminus of Pik1 used in that study may have interfered with its localization. Consistent with this, Pik1-GFP as a C-terminal fusion in *S. cerevisiae* is stable but not functional [14]. Our observation of Pik1 medial localization required the use of a 2XeGFP-Pik1 fusion protein in *Δpik1::ura4* cells. The fusion protein provided all essential functions, as cell proliferation and morphology were similar to wild-type. The appearance of Pik1 at the medial region corresponded to the time of septum material deposition, and was clearly after the onset of mitosis and formation of the F-actin ring (Figure 4).

*S. cerevisiae* Pik1 is found in the nucleus in addition to the Golgi, although its biological function in the nucleus is uncertain [14]. A nuclear localization of Pik1 in *S. pombe* was not observed in this study or in a global localization study [18]. It is possible that a nuclear pool of Pik1 is small and not detected under our experimental conditions. However, our complementation study in which expression of an *S. pombe pik1* cDNA provided the essential functions that were lost in an *S. cerevisiae pik1-101* strain may suggest that *S. pombe* Pik1 is capable of providing essential nuclear functions. The *pik1-101* mutation is a serine to phenylalanine substitution at residue 1045. This residue is within the kinase domain as identified by Prosite (http://ca.expasy.org/prosite/). The *pik1-101* allele appears to be either kinase-dead or greatly reduced for kinase activity [7]. Pik1 lipid kinase acitivity is required in both the Golgi and in the nucleus in *S. cerevisiae* [14]. Our observation of complete complementation suggests that either the fission yeast protein is reaching the nucleus in the budding yeast or that the *pik1-101* strain possesses sufficient residual kinase activity in the nucleus at the restrictive temperature.

### Pik1 is required for septation

Consistent with the timing of its appearance at the division site (Figure 4), loss of Pik1 function in the *pik1-td* strain is associated with failure of processes in late cytokinesis (Figures 2 and 3). Most obvious were the observations of supernumerary septa, the accumulation of excessive amounts of secondary septum material and the persistence of the primary septum (Figures 2 and 3). Thus, Pik1 activity may be required to signal the termination of septum material deposition, to initiate the hydrolysis of the primary septum or to suppress reinitiation of septation. The observations noted above may result from loss of Pik1-dependent Golgi functions or from loss of Pik1 activity at the medial region, or both. The importance of the lipid kinase activity of Pik1 was suggested by the observation that ectopic expression of the kinase-dead allele *pik1^D709A^* also resulted increased septation index and failure of cell separation (Figure 8 and Table 3). Examination of the phenotype of cells carrying the chromosomally integrated kinase-dead allele revealed that its kinase activity is essential for vegetative growth (Figure 1). The observation of an elongated cell, terminal phenotype by tetrad analysis suggested a cytokinesis defect. Ectopic expression of *pik1^D709A^* resulted in a dominant lethal phenotype with partial penetrance compared to that produced by ectopic expression of the wild-type allele (Table 3). While actin structures, including the contractile ring, appeared to be normal, defects in septation were observed. Thus, Pik1 lipid kinase activity is required for septation.

Two possible functions of Pik1, its lipid kinase activity and its Cdc4-binding activity, were examined in this study. We identified Pik1 residues, D709 and R838, that are required for its lipid kinase activity and for its interaction with Cdc4, respectively. Our initial approach was to attempt to create a *pik1* kinase-null allele and by ectopic expression of that allele to disrupt the activity of the endogenous enzyme. As described above, this approach provided evidence for an essential function of Pik1 lipid kinase activity in cell division. Similarly, we hoped to create an allele that was impaired for binding to Cdc4 and to use ectopic expression of that allele to identify functions related to the Pik1-Cdc4 protein interaction. Integration of this allele into the chromosomal *pik1* locus was then used to assess the cell phenotypes when expressed under the control of the native promoter.

Control experiments revealed that ectopic expression of wild-type *pik1* in cells that were otherwise wild-type for *pik1* and *cdc4*, produced a dominant lethal phenotype. The terminal phenotype revealed disruption of the actin cytoskeleton structure, including very few cells with contractile rings (Table 3). In stark contrast, ectopic expression of the double mutant allele, *pik1^D709A, R838A^*, was apparently innocuous. Thus, the dominant lethal phenotype can be attributed fully to Pik1 activities that depend on the two substituted residues. The observed lethal phenotypes associated with ectopic expression of some *pik1* alleles cannot be attributed solely to the increased levels of lipid kinase activity that we observed in cell lysates. As an example, cells in which *pik1* or *pik1^R838A^* were ectopically expressed both accumulated elevated levels of the ectopic protein (Figure 6) and of lipid kinase activity (Figure 7); yet, the former died while the latter were viable (Figure 8). Examination of the phenotype of cells carrying the chromosomally integrated *pik1^R838A^* substitution revealed that R838 is non-essential for vegetative growth. Cells expressing only this allele were phenotypically wild-type. Notwithstanding, the R838 residue appears to be important for Pik1 functions. Ectopic expression of *pik1^R838A^* was almost innocuous (Figure 8). Since ectopically expressed Pik1^R838A^ accumulated and was active as a lipid kinase we can infer that this mutation does not cause folding or stability problems for the protein. These observations demonstrate that the dominant lethal phenotype associated with ectopic expression of *pik1* is largely attributable to a function requiring the R838 residue. One such function may be an interaction between Pik1 and Cdc4, as suggested in this work based on yeast two-hybrid and ELISA studies. Also consistent with this hypothesis is the observation that the lethal phenotype associated with ectopic expression of wild-type *pik1* was almost completely suppressed in *cdc4^G107S^* cells (Figure 9). We had shown previously that the G107 residue of Cdc4 is required for the interaction between Cdc4 and the C-terminal 345 amino acids of Pik1 [21]. A peptide spanning the pseudo IQ motif of Pik1 including the R838 residue has also been shown to bind purified Cdc4 by NMR spectroscopy (Escobar-Cabrera *et al*., personal communication). The interaction between Pik1 and Cdc4 may be functionally important; however, whether it affects the activity or localization of Pik1 is unknown.

Overall, these results suggest that Pik1 is a lipid kinase that is recruited to the medial cell plane late in cytokinesis and is required for septation. These results also suggest that protein-protein interactions involving the R838 residue of Pik1, possibly with Cdc4, are functionally significant, although perhaps not essential.
